# Distinguishing Oligosaccharide Isomers Using Far-Infrared Ion Spectroscopy: Identification of Biomarkers for Inborn Errors of Metabolism

**DOI:** 10.1021/acs.analchem.3c00363

**Published:** 2023-06-21

**Authors:** Rianne E. van Outersterp, Pieter C. Kooijman, Jona Merx, Udo F.H. Engelke, Nematollah Omidikia, Mei-Lan H. Tonneijck, Kas J. Houthuijs, Giel Berden, Tessa M.A. Peters, Dirk J. Lefeber, Michel A. A. P. Willemsen, Jasmin Mecinovic, Jeroen J. Jansen, Karlien L.M. Coene, Ron A. Wevers, Thomas J. Boltje, Jos Oomens, Jonathan Martens

**Affiliations:** † Institute for Molecules and Materials, FELIX Laboratory, Radboud University, 6525 ED Nijmegen, The Netherlands; ‡ Institute for Molecules and Materials, Synthetic Organic Chemistry, Radboud University, 6525 AJ Nijmegen, The Netherlands; § Department of Laboratory Medicine, Translational Metabolic Laboratory, Radboud University Medical Centre, 6525 GA Nijmegen, The Netherlands; ∥ Department of Analytical Chemistry and Chemometrics, Institute for Molecules and Materials, Radboud University, 6525 AJ Nijmegen, The Netherlands; ⊥ Department of Neurology, Donders Institute for Brain, Cognition and Behaviour, Radboud University Medical Centre, 6500 HB Nijmegen, The Netherlands; # Amalia Children’s Hospital, Department of Pediatric Neurology & Donders Institute for Brain, Cognition and Behaviour, Radboud University Medical Centre, 6500 HB Nijmegen, The Netherlands; ∇ Department of Physics, Chemistry and Pharmacy, University of Southern Denmark, 5230 Odense, Denmark; ○ Department of Clinical Chemistry and Hematology, Elisabeth-TweeSteden Hospital, 5042 AD Tilburg, The Netherlands; ◆ van’t Hoff Institute for Molecular Sciences, University of Amsterdam, 1098 XH Amsterdam, The Netherlands

## Abstract

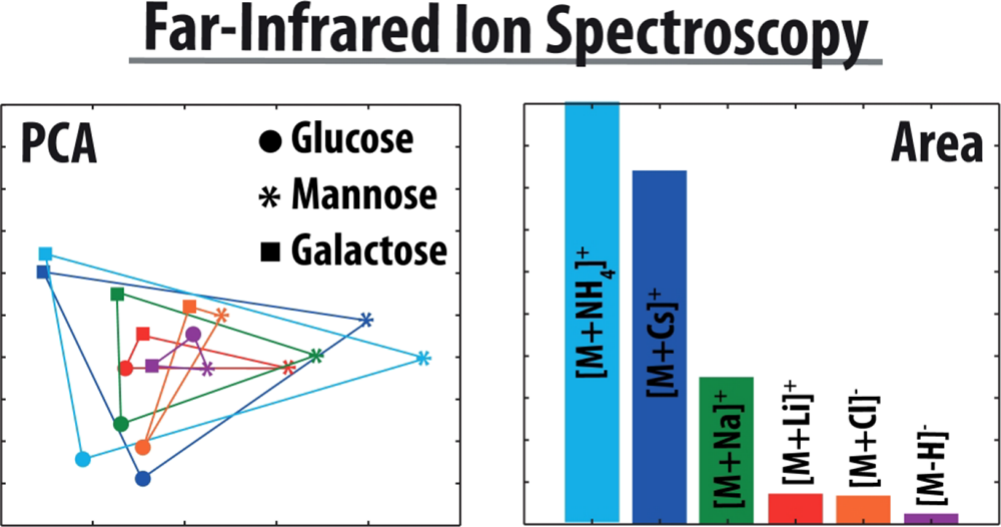

Distinguishing isomeric saccharides poses a major challenge for analytical workflows based on (liquid chromatography) mass spectrometry (LC–MS). In recent years, many studies have proposed infrared ion spectroscopy as a possible solution as the orthogonal, spectroscopic characterization of mass-selected ions can often distinguish isomeric species that remain unresolved using conventional MS. However, the high conformational flexibility and extensive hydrogen bonding in saccharides cause their room-temperature fingerprint infrared spectra to have broad features that often lack diagnostic value. Here, we show that room-temperature infrared spectra of ion-complexed saccharides recorded in the previously unexplored far-infrared wavelength range (300–1000 cm^–1^) provide well-resolved and highly diagnostic features. We show that this enables distinction of isomeric saccharides that differ either by their composition of monosaccharide units and/or the orientation of their glycosidic linkages. We demonstrate the utility of this approach from single monosaccharides up to isomeric tetrasaccharides differing only by the configuration of a single glycosidic linkage. Furthermore, through hyphenation with hydrophilic interaction liquid chromatography, we identify oligosaccharide biomarkers in patient body fluid samples, demonstrating a generalized and highly sensitive MS-based method for the identification of saccharides found in complex sample matrices.

## Introduction

While untargeted metabolic profiling based on liquid chromatography-mass spectrometry (LC–MS) is frequently used in the search for small-molecule biomarkers in clinical biochemistry, the chemical identification of detected *m/z* features remains a fundamental limitation.^1,2^ An example is the field of inborn errors of metabolism (IEMs), where untargeted LC–MS is now the most widely used technique to discover metabolites whose levels (accumulated or depleted) in body fluids correlate with a particular disease.^3,4^ Although this approach has led to many breakthroughs in the field, both in terms of our understanding of disease pathophysiology and in aiding development of targeted diagnostic protocols, metabolite identification often remains prohibitively costly and time-consuming.

While saccharides and saccharide-derivatives play a central role in biology and may therefore serve as biomarkers for a range of conditions,^5^ they are also particularly challenging for routine LC–MS protocols. Individual isomeric monosaccharide building blocks differ by their stereochemistry at one or multiple chiral centers and can be linked in regio-isomeric and stereo-isomeric ways to form oligosaccharides. Moreover, isomeric oligosaccharides are difficult to separate by LC–MS and typically have very similar MS/MS fragmentation patterns. In order to address this challenge, several specialized approaches have been developed, distinguishing saccharides based on subtle differences in relative MS^*n*^ fragment ion intensities,^6^ chemical derivatization prior to MS/MS analysis,^7^ rates of water adduct formation of lithiated saccharides,^8^ MS/MS using a wavelength-tuneable CO_2_ laser for photodissociation,^9^ or dissociation rates of metal-bound clusters of saccharides and chiral ligands.^10−12^ However, these methods are often impractical for application to complex biochemical samples and/or require reference standards of all candidates for reliable identification, making them better suited for targeted assays (which also holds true for methods relying on chromatographic retention times).^13^ In recent years, ion mobility spectrometry (IMS) has also been increasingly used for saccharide identification.^14,15^ An advantage of IMS is that collisional cross sections can be predicted computationally. However, correlating a predicted value to an experimentally measured one is often challenging as the CCS is a single value in contrast to a spectroscopic fingerprint. Additionally, in some cases, IMS can be performed on MS/MS fragment ions in a bottom-up structural elucidation approach.

Alternatively, several recent studies have used various forms of infrared ion spectroscopy (IRIS), which provides *m/*z-selective IR spectra and therefore structural information of gas-phase ionic saccharides trapped in an ion storage mass spectrometer.^16−28^ Approaches based on room temperature infrared multiple photon dissociation (IRMPD) are attractive because they can be implemented on commercial MS platforms and can be directly integrated with existing LC–MS workflows. However, due to their high conformational flexibility, IR spectra of saccharides recorded at room temperature in the IR fingerprint region often contain relatively broad spectral bands. This causes IR spectra of saccharide isomers, especially when unfunctionalized, in many cases to be relatively similar and unsuitable as a diagnostic tool.^16^ Cryogenic ion spectroscopy offers higher IR spectral resolution and can more reliably discriminate saccharides.^29−31^ However, these experiments are currently based on home-built instrumentation that is difficult to incorporate in the LC–MS based workflows regularly used for experiments in a clinical context (though a few examples do exist).^22,23,32^

Unfunctionalized saccharides have relatively low proton affinities and are mostly detected as ammonium, potassium, or sodium adducts rather than protonated ions in positive mode electrospray ionization (ESI)-LC–MS. In negative mode, deprotonated ions and chloride adducts are typically observed.^33^ The many hydroxyl groups offer strong interaction sites for alkali metals,^34^ where the alkali ion-saccharide bond strength increases with decreasing ionic radius of the metal. Therefore, (direct infusion) fragmentation MS studies differentiating saccharides focus on Li^+^-adducts as the strong binding leads to rich intramolecular fragmentation rather than expulsion of the alkali cation, as is usually observed for larger cations (especially K^+^, Rb^+^, and Cs^+^). In contrast, complexes with larger alkali metals are appealing for IRIS studies since the metal ion acts as a dissociation tag because of its lower dissociation threshold. Alkali ion complexation of saccharides has also been exploited in IRIS studies to increase the spectral contrast between isomeric structures.^19,20,35^ Complexation with cations or anions locks in the conformation and affects the hydrogen bonding pattern of saccharides, which is specific to individual isomeric structures. These changes in the three-dimensional structure may affect the positions of vibrational resonances monitored by IRIS.

IR spectra of small saccharides and saccharide derivatives recorded at room-temperature using IRIS have been reported in the IR fingerprint region (900–1800 cm^–1^ in most studies). These spectra generally have broad IR features that have minimal diagnostic value, especially for saccharides without functionalization (such as *N*-acetyl, phosphate or sulphate groups).^16,17,36,37^ IR spectra in the OH/NH stretching region (3000–3700 cm^–1^) contain more isolated bands and have been used in numerous studies to differentiate isomers.^18−20,24,25,35,38−40^ In contrast, the long-wavelength region of the IR spectrum (300–1000 cm^–1^), containing more delocalized vibrations, has thus far been largely neglected for saccharide identification.

Here, we introduce an approach using IRIS in the far-infrared region targeting several types of saccharide ions. We compare the IR spectra of different types of adduct ions in this wavelength range and show that Cs^+^, Na^+^, and NH_4_^+^ complexes with oligosaccharides have well-resolved, sharp, and highly diagnostic vibrations. Here, Cs^+^ behaves like an ion tag, which is readily dissociated at moderate irradiation power. On the other hand, Na^+^ and NH_4_^+^ complexes are the ions most often detected in analytical LC–MS workflows. Extending the method to larger systems, we demonstrate that the method discriminates oligosaccharides up to tetrasaccharides that differ only by the configuration of a single glycosidic linkage. For identification of saccharides directly from complex samples (body fluids), hyphenation with hydrophilic interaction liquid chromatography (HILIC) was implemented. We show the identification of several metabolites that serve as biomarkers for IEMs, including two novel biomarkers that we identified using this approach.

## Methods

### LC–MS Experiments

Sample preparation and LC–MS methods used in this study have been reported previously^41,42^ and a more detailed description and references can be found in the Supporting Information.

### Infrared Ion Spectroscopy

IRIS experiments were performed in a quadrupole ion trap mass spectrometer (Bruker, amaZon Speed ETD) modified for spectroscopy using the FELIX free electron laser. Details of the hardware modifications and synchronization of the experiment are described elsewhere.^43^ Coupling of the LC–MS experiments with the IRIS experiments is described elsewhere^42^ and the precise implementation employed here is described in more detail in the Supporting Information.

### PCA, Cosine Similarity Scoring, and Peak Picking

In the principal component analysis (PCA),^44^ each spectrum was interpolated with a resolution of 1 cm^–1^. PCA was conducted using the MATLAB 2021a (MathWorks, Natick, MA, USA) software package. The data were group mean centered per ion-type. and each spectrum was normalized to the maximum intensity. For the PCA in Figure 4, IR spectra of patient samples were projected to the PCA space. The similarity between patient IR spectra and reference spectra was quantified by calculating the Euclidean distance (ED) between data points via
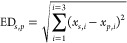
Here, *x_s,i_* and *x_p,i_* are the *i*^th^ coordinates of the reference and the patient sample spectra in the PCA plot, respectively.

To calculate the cosine similarity (*S*(*A*,*B*)) between pairs of spectra *A* and *B*, the spectra were interpolated in the 305–990 cm^–1^ range using a resolution of 1 cm^–1^. The cosine similarity was defined as
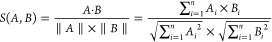
To quantify the number of features in the far-IR and fingerprint IR ranges we applied the *peakfinds* function as implemented in the MATLAB 2021a (MathWorks, Natick, MA, USA) software package. The IR spectra were smoothed using a Savitky-Golay filter. Here, the degree of the polynomial was 4, and the length of the sliding window was 45. The minimum peak height and the peak distances were set as 0.070 and 15, respectively.

## Results and Discussion

### Monosaccharides

We selected three unfunctionalized diastereomeric aldohexoses, d-glucose, d-galactose and d-mannose, common monosaccharides playing central roles in human metabolism. Their structures differ in the stereochemistry of the OH-groups: d-galactose is the C4- and d-mannose is the C2-epimer of d-glucose, respectively. They are found as a mixture of their alpha- and beta-pyranose forms in an ESI-MS experiment.

**Figure 1:**
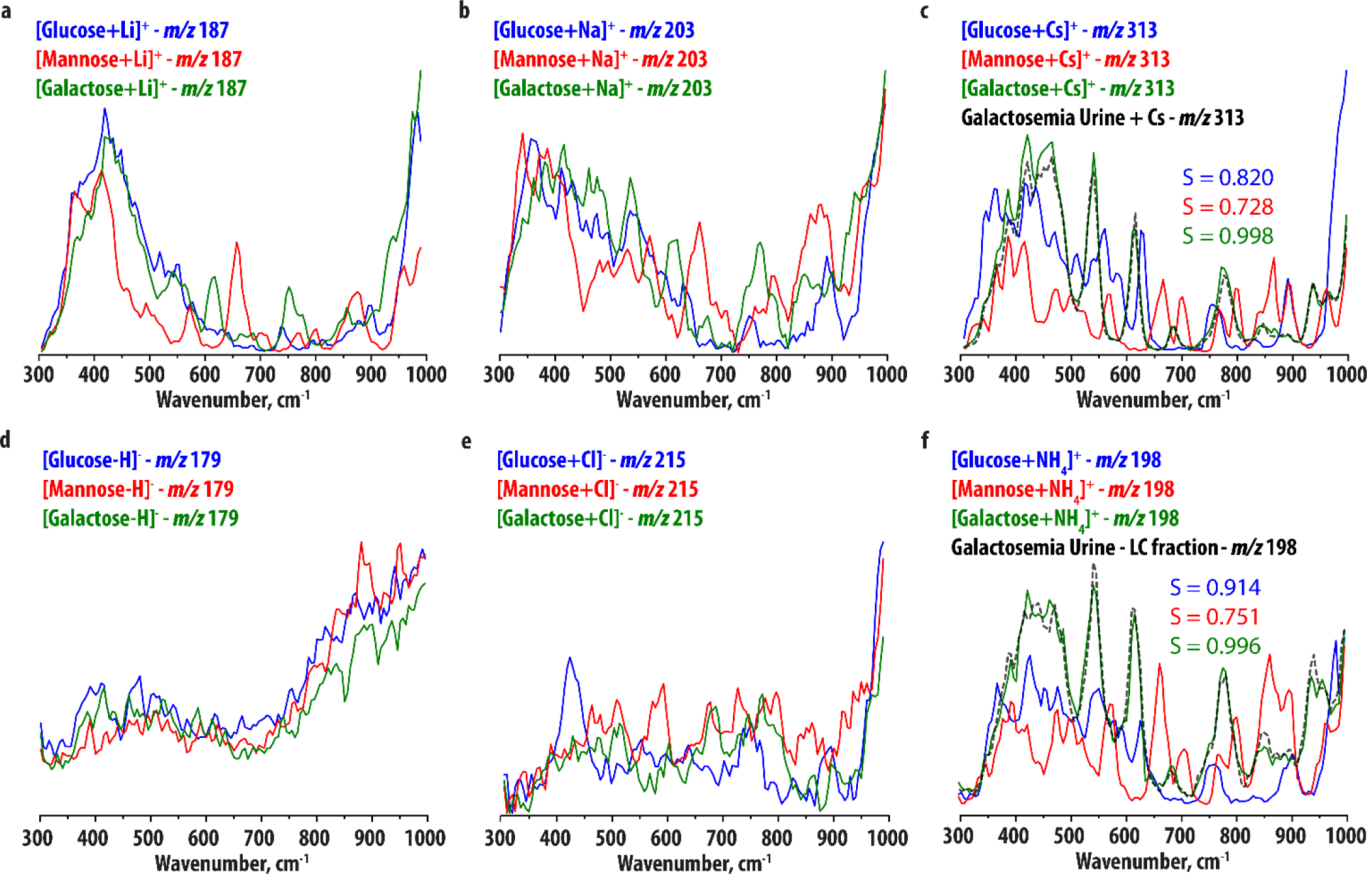
Comparison of the experimental IR spectra of the (a) [M+Li]^+^, (b) [M+Na]^+^, (c) [M+Cs]^+^, (d) [M–H]^−^, (e) [M+Cl]^−^, and (f) [M+NH_4_]^+^ ions of d-glucose (blue), d-mannose (red), and d-galactose (green). Panels (c) and (f) compare the IR spectra of the *m/z* 313 ion ([M+Cs]^+^) detected in cesium-spiked urine of a galactosemia patient and the *m/z* 198 ion ([M+NH_4_]^+^) detected in the HILIC-MS analysis from the same (un-spiked) urine sample (black traces) to the three reference spectra for the same ion-type.

We recorded IR spectra of the Li^+^, Na^+^, and Cs^+^-adducts of d-glucose, d-mannose, and d-galactose using IRIS. As expected, photofragmentation MS/MS spectra of the Li^+^-adducts show rich intramolecular (ring) fragmentation due to the higher binding strength of the cation (see Figure S1). Conversely, dissociation of the Na^+^ and Cs^+^-adducts proceeds almost exclusively via the loss of the cation, leaving the intact saccharide structure as the neutral loss. This is in line with previous studies.^45^ The IR spectra of the Na^+^-adducts were recorded as precursor ion depletion spectra (see Extended Methods section in the Supporting Information) since Na^+^ cannot be detected in the ion trap at *m/z* 23 (below the low-mass cut-off). In contrast, spectra of Cs^+^-adducts could be recorded as fragmentation yield spectra. IR spectra are presented in Figure 1a–c, and all types of metal-adducts show sufficient differences to allow differentiation of the sugars. On the other hand, the IR spectra of the different metal ion adducts of the same sugar generally contain similar features (see Figure S2 for direct overlays), suggesting that the different cations have a broadly similar influence on the conformation and hydrogen-bonding patterns of these hexoses. In general, the Cs^+^ spectra contain more and better-defined features compared to the Li^+^ spectra, likely as a consequence of more resonances reaching internal energies above the threshold for dissociation in this more weakly-bound complex. Note, for example, the peak at ∼700 cm^–1^ in the spectrum of [d-Mannose + Cs]^+^, which has a low intensity in the spectrum of [d-Mannose + Li]^+^. The IR spectra of the Na^+^-adducts show more similarity with the IR spectra of the Cs^+^-adducts but have significantly poorer signal-to-noise ratios since they are measured as ion depletion spectra, which are inherently sensitive to variations in the total ion count. Therefore, we conclude that of the three cations tested here, Cs^+^ is best suited for monosaccharide identification in this wavelength range.

Note that the differentiating spectral features in Figure 1c are not only small variations in the relative band intensities, but well-resolved spectral bands. As a comparison, we recorded IR spectra of the [M+Cs]^+^ ions of the three hexoses in the fingerprint range (see Figure S3). These spectra do not show well-resolved bands, and the hexoses are not straightforwardly distinguished on this basis. To quantify this, we calculated the cosine similarity between the [M+Cs]^+^ spectra in both the far IR (defined here as 310–995 cm^–1^) and fingerprint IR (defined here as 900–1845 cm^–1^) wavelength regions. The cosine similarity score (see Method section) is a value between 0 and 1 that is a measure of similarity between two vectors. Here, a value of 1 means that the vectors are identical whereas a value of 0 means that the vectors are completely orthogonal. In the far-IR range, we obtained cosine similarity scores of 0.776 (for d-Glucose and d-Mannose), 0.866 (d-Glucose and d-Galactose), and 0.732 (d-Mannose and d-Galactose). In contrast, the scores in the fingerprint range were 0.963, 0.974, and 0.975 for the three sets respectively, confirming that the spectra are more similar in this wavelength range. To quantify the number of discriminative features in the IR spectra, we performed a peak-picking algorithm (see Methods section and Figure S4 and Table S1 for details) on each of the [M+Cs]^+^ spectra in both the far-IR and fingerprint wavelength regions. This resulted in 10, 13, and 10 unique peaks (with an average full width at half maximum (FWHM) of 31 cm^–1^) in the far-IR range and 4, 4, and 6 unique peaks (with an average FWHM of 66 cm^–1^) in the fingerprint range for d-Glucose, d-Mannose, and d-Galactose, respectively.

To demonstrate the analytical applicability of the method to a biological sample, we selected a urine sample from a patient with classic galactosemia, an IEM caused by a deficiency of Galactose-1-phosphate uridyltransferase, which results in the accumulation of d-galactose in urine. The sample preparation procedure consisted only of dilution, centrifugation, and spiking with a solution of CsCl to produce the Cs^+^-adduct of the accumulated saccharide. Direct infusion ESI was employed. Figure 1c compares the IR spectrum recorded for the *m/z* 313 peak in the spiked urine sample to the three [hexose + Cs]^+^-reference spectra, showing a clear spectral match with d-galactose. The cosine similarity score between the *m/z* 313 spectrum measured from the urine sample and the three reference IR spectra support the assignment of the d-galactose structure.

Urine of galactosemia patients contains high concentrations of d-galactose (quantified in the sample used in this study at 18,000 μmol mmol^–1^ creatinine, see Methods section), reducing the chance of isomeric compounds interfering with the analysis (for example, normal range of urinary d-glucose is 10–278 μmol mmol^–1^ creatinine^46^). However, in most cases a separation prior to IRIS analysis is required when analyzing biochemical samples to obtain maximum sensitivity and to prevent measurement of an IR spectrum of a mixture of isobaric species. High-pressure LC (HPLC) is often the separation method of choice, and its coupling to IRIS has been demonstrated in several studies.^42,47−49^ However, most MS-compatible mobile phases in LC do not directly support the formation of saccharide Cs^+^-adducts, but rather Na^+^, K^+^, NH_4_^+^, and Cl^–^-adducts (as these are common additives or contaminant ions) and deprotonated ions. Post-column addition of metal salts to support adduct formation in MS studies has been demonstrated, but is cumbersome in practice for routine IRIS analysis and would likely reduce the sensitivity. Hence, to explore the feasibility of identifying saccharide adducts using direct coupling of LC with IRIS, we recorded IR spectra of the deprotonated ions and NH_4_^+^ and Cl^–^-adducts of d-glucose, d-mannose, and d-galactose (K^+^-adducts give rise to ion depletion spectra, see Na^+^-adducts above). The resulting spectra are presented in Figure 1d–f. Photofragmentation of the Cl^–^-adducts proceeds via loss of the Cl^–^ anion (see Figure S1 for photofragmentation spectra) so that these adducts can only be measured as precursor ion depletion spectra. For the deprotonated systems, a rich fragmentation pattern was observed, but the IR spectra show broadened spectral bands with minimal diagnostic value, likely due to shared-proton motifs in saccharide anions. The NH_4_^+^-adducts on the other hand have well-resolved IR spectra that easily allow us to differentiate the monosaccharides, providing an excellent alternative to Cs^+^-cations in this wavelength range. Interestingly, the IR spectra of the NH_4_^+^ and Cs^+^-adducts are very similar (see Figure S5 for a direct overlay), suggesting that the conformation and hydrogen bonding pattern of the hexoses and their interaction with the cation is similar for the two adduct ions.

**Figure 2:**
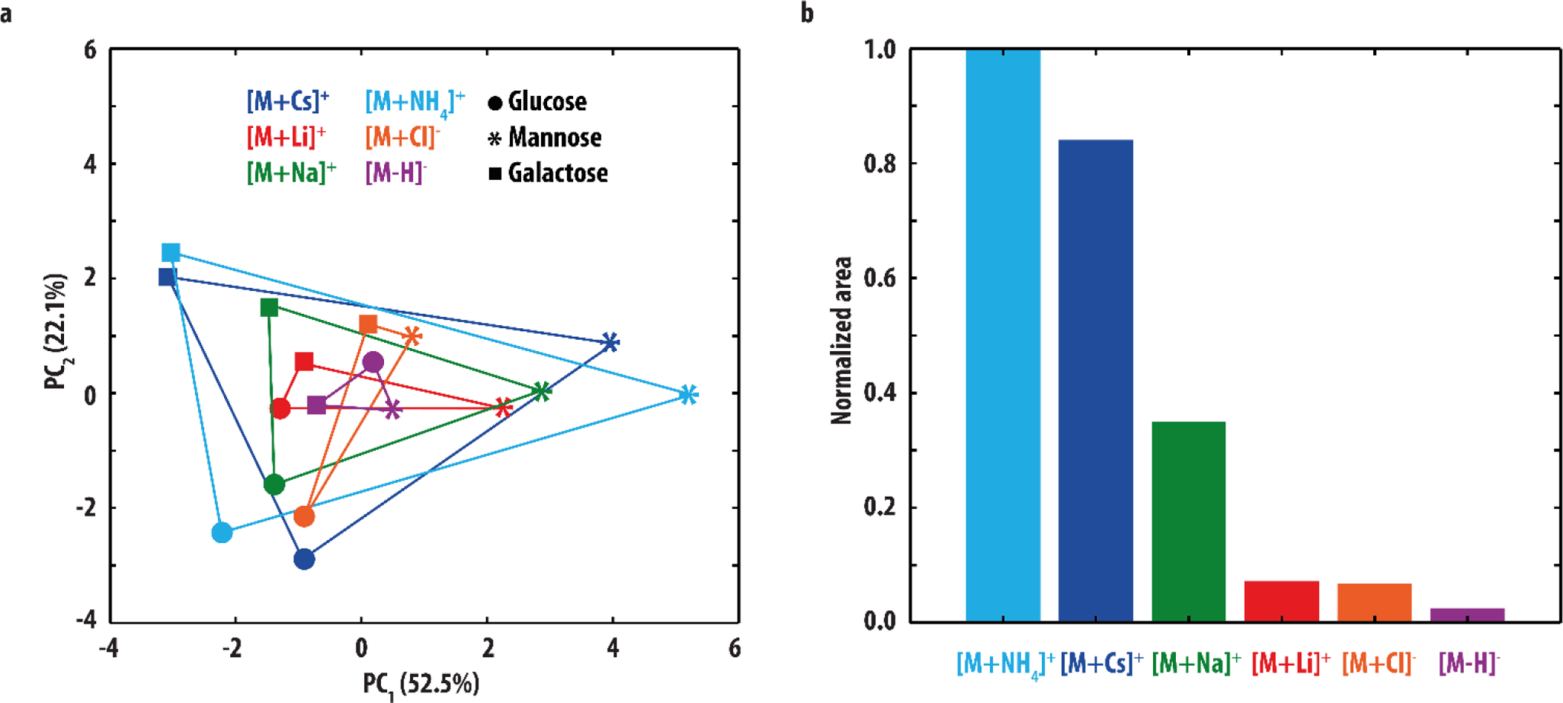
(a) Score plot resulting from the PCA of the IR spectra of the [M+Cs]^+^ (blue), [M+Li]^+^ (red), [M+Na]^+^ (green), [M+NH_4_]^+^ (light blue), [M+Cl]^−^ (orange), and [M–H]^−^ (purple) ions of d-glucose (circles), d-mannose (stars) and d-galactose (squares). A smaller distance between two points correlates with greater similarity between two spectra. (b) Normalized surface areas enveloped by the triangles drawn in (a), which are a measure for the degree of variation in each set of hexose spectra.

To quantify the degree to which the various adduct-types are able to differentiate between saccharides, we performed a PCA^44^ on the IR spectra of all hexose ions. Here, a multidimensional data-set is reduced to fewer dimensions by defining principal components, new orthogonal variables that best describe the variation within the data. The PCA resulted in two PCs describing 74.6% of the data. The sum-of-squares of the residuals was equal to 43.7 out of a total variance of 172.48 for the data set (=25.3%). Plotting the IR spectra in this new two-dimensional space (Figure 2a) illustrates the degree of variation between the IR spectra, where a larger distance between two data-points indicates greater distinction. Figure 2a shows that IR spectra of different adducts of the same hexose are in general grouped together: the d-glucose spectra are found in the bottom-left corner, d-mannose on the right and d-galactose in the top-left corner, confirming that these IR spectra are very similar (as observed above). The surface area of the triangles connecting the three hexose spectra of the same adduct-ion relates to the variation between these spectra. Figure 2b compares the surface areas for each adduct-type, confirming that the NH_4_^+^- and Cs^+^-adducts give the best discrimination between the hexoses. Note that for some saccharides, the dissociation energy of the NH_4_^+^ adduct may be higher than the Cs^+^ adduct, preventing the complexes from reaching internal energies above the dissociation threshold at some absorption bands and thus causing them to be absent in the recorded IR spectrum. Where analytically possible, Cs^+^-adducts are therefore still the preferred option.

HILIC retains highly polar compounds and uses mobile phases compatible with MS, so it is often the method of choice for the LC–MS analysis of saccharides. We developed a HILIC protocol using an amide column and a mobile phase containing ammonium hydroxide (see Methods section) to favor the formation of ammonium adducts. Separation of the galactosemia urine sample produces a feature at *m/z* 198 eluting between 6.85–7.05 min, and this fraction of eluent was analyzed by IRIS. Figure 1f compares the IR spectrum recorded for the *m/z* 198 peak in this fraction with the three [hexose + NH_4_]^+^ reference spectra, showing a clear match to the d-galactose-NH_4_^+^ IR spectrum and indeed a favorable cosine similarity score.

### Disaccharides

**Figure 3:**
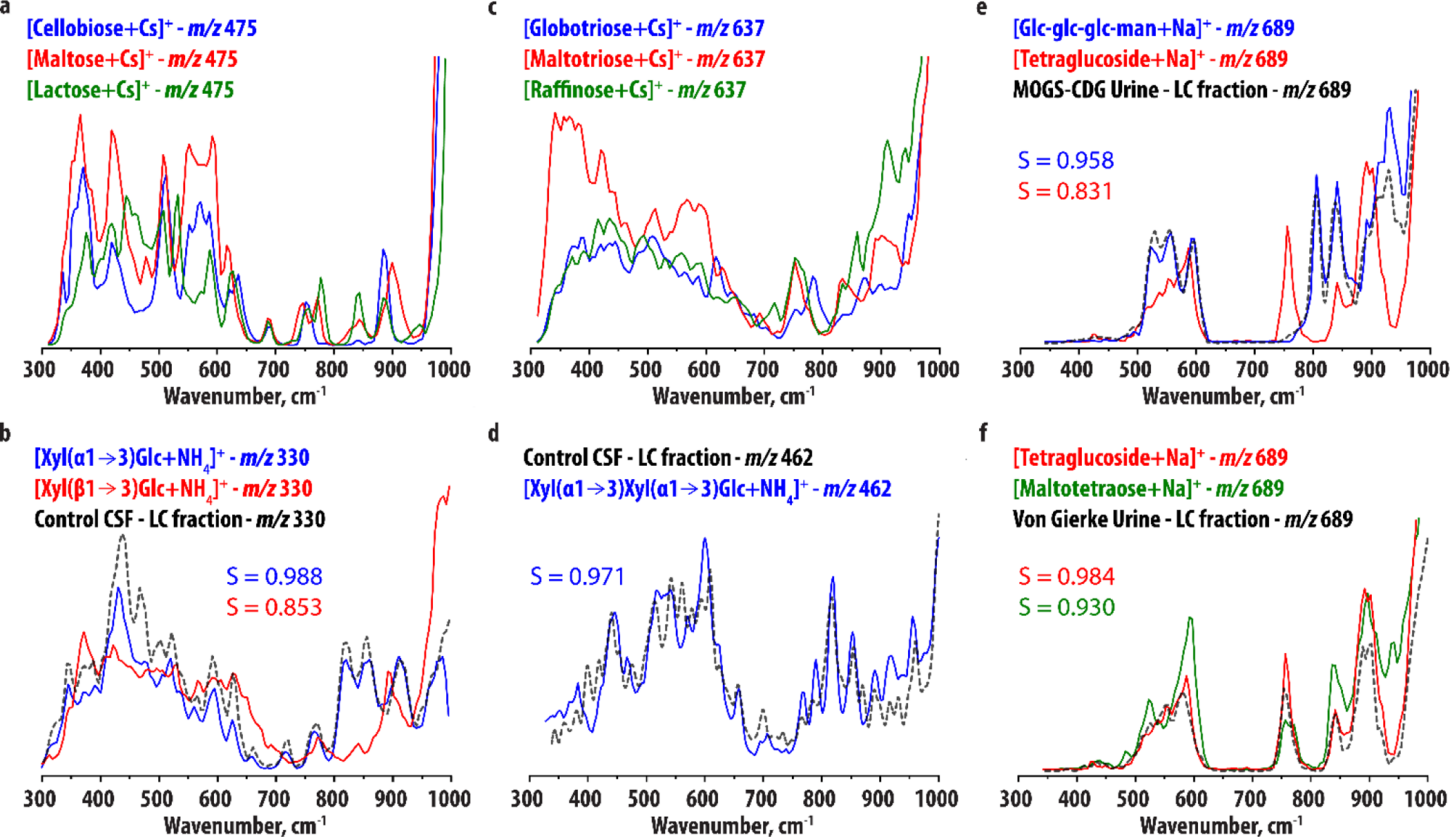
(a, b) Comparison of the experimental IR spectra of (a) [M+Cs]^+^ ions of d-cellobiose, d-maltose, and d-lactose and (b) [M+NH_4_]^+^ ions of Xyl(α1→3)Glc and Xyl(β1→3)Glc. Panel (b) compares the IR spectrum of the *m/z* 330 ion ([M+NH_4_]^+^) detected in the HILIC-MS analysis of a CSF sample (black trace) to the two reference spectra. (c) Comparison of the experimental IR spectra of the [M + Cs]^+^ ions of d-globotriose (blue), d-maltotriose (red), and d-raffinose (green). (d) IR spectrum of the *m/z* 462 ion ([M+NH_4_]^+^) detected in the HILIC-MS analysis of a CSF sample (black trace) in comparison to the experimental IR spectrum of the [M+NH_4_]^+^ ion of Xyl(α1→3) Xyl(α1→3)Glc (blue trace). (e) IR spectrum of the *m/z* 689 ion ([M+Na]^+^) detected in the HILIC-MS analysis of a urine sample of a MOGS-CDG patient (black trace) in comparison to the experimental IR spectra of the [M+Na]^+^ ions of Glc-Glc-Glc-Man (blue trace) and d-tetraglucoside (red). (f) IR spectrum of the *m/z* 689 ion ([M+Na]^+^) detected in the HILIC-MS analysis of a urine sample of a Von Gierke disease patient (black trace) in comparison to the experimental IR spectrum of the [M+Na]^+^ ions of d-tetraglucoside (red) and d-maltotetraose (green).

While we used these simple isomeric hexoses to optimize and validate our approach, the distinction of larger isomeric oligosaccharides is a greater challenge since these may not only differ in their monosaccharide constituents but also in the stereochemistry of their glycosidic linkages. With increasing molecular size, IR spectra tend to become more congested, which may mask the differences between closely related isomers. This especially hampers IRMPD spectroscopy at room temperature. To explore the limits of our approach, we recorded IR spectra of larger oligosaccharide structures. Figure 3a compares the recorded IR spectrum of the Cs^+^ adduct of the disaccharide d-cellobiose (Glc(β1→4)Glc) to the IR spectra of d-maltose(Glc(α1→4)Glc), which differs in the stereochemistry of the glycosidic linkage, and d-lactose (Gal(β1→4)Glc), which has a galactose unit at the first position instead of glucose. These IR spectra show well-defined and sharp vibrational features and the saccharide isomers can be distinguished based on well-resolved frequency differences. Note that d-cellobiose and d-lactose share a vibrational band at 885 cm^–1^, which is blue-shifted in the d-maltose spectrum, suggesting that this band is sensitive to the linkage stereochemistry. d-cellobiose and d-maltose, on the other hand, show similarity in the 300–650 cm^–1^ region, suggesting that this region contains more information on the monosaccharide constituents.

As described above and in the Methods section, we combined HILIC and IRIS of NH_4_^+^-adducts to identify novel biomarkers for glucose transporter type 1 deficiency syndrome (GLUT1DS), an IEM characterized by neuroglycopenia (low brain glucose levels) leading to intellectual disability, movement disorders, and drug-resistant epilepsy.^50^ We focus here on the IR spectroscopy, but a full description of the detection of the metabolites and the implications for the pathophysiological understanding of GLUT1DS is available separately.^51^ Untargeted metabolomics recognized three *m/z*-values with decreased intensity in the cerebrospinal fluid (CSF) of patients with GLUT1DS compared to controls. Based on accurate mass measurements and the biochemical context of the disease, one of the features was proposed to be a Xyl-Glc disaccharide. On the basis of MS/MS alone, the stereochemistry of the linkage between the monosaccharide units could not be determined. Therefore, we synthesized reference standards for Xyl(α1→3)Glc and Xyl(β1→3)Glc (see Supporting Information) and recorded the IR spectra of their NH_4_^+^-adducts. The comparison in Figure 3b shows that the two disaccharides are easily distinguished, most notably in the 700–950 cm^–1^ range. Again, the band around 900 cm^–1^ is blue-shifted for the α-isomer, as for the other disaccharides, further suggesting that this band is diagnostic for the linkage stereochemistry. Figure 3b contains the comparison of the IR spectrum of the *m/z* 330 ion detected in HILIC-MS analysis of CSF of controls to the two reference spectra, indicating the unknown is Xyl(α1→3)-Glc. The cosine similarity score inlayed in the figure supports this assignment. Note that the concentration of the disaccharide in CSF is low (<10 nM), which may explain the small deviations in intensities. This also explains the slightly lower cosine similarity score (0.988) between the spectrum of the unknown and the Xyl(α1→3)-Glc spectrum as compared to the cosine similarity scores reported for the galactose ions above (0.998 and 0.996).

### Tri- and Tetrasaccharides

Figure 3c compares the IR spectra of the Cs^+^-adducts of d-globotriose (Gal(α1→4)Gal(β1→4)Glc), d-Maltotriose (Glc(α1→4)Glc(α1→4)Glc), and d-raffinose (Gal(α1→6)Glc(α1→2)β-Fru): three isomeric trisaccharides differing in their monosaccharide constituents and linkages. The IR spectra are more congested than the monosaccharide and disaccharide spectra, but retain sufficient diagnostic value to distinguish the three systems. Note the shifted band around 800 cm^–1^ for d-globotriose, the different band patterns for the three systems in the 800–950 cm^–1^ range, and the distinctive shape of the 300–700 cm^–1^ range for d-maltotriose. d-globotriose contains a β-linkage and has a band just below 900 cm^–1^, whereas the other systems (containing exclusively α-linkages) have a band above 900 cm^–1^, consistent with the suggestion that this vibration is diagnostic for linkage stereochemistry.

In addition to the disaccharide discussed in the previous section, another *m/z* value differentiating GLUT1DS patient CSF from control CSF was recognized to likely be a trisaccharide. Based on biochemistry,^51^ we hypothesized it to be Xyl(α1→3)Xyl(α1→3)Glc. Figure 3d compares the IR spectrum of the metabolite to the IR spectrum of a synthesized reference standard. As there were no alternative reference standards available, we used multiple analytical techniques to assign the structure.^51^ The Xyl(α1→3)Xyl(α1→3)Glc reference (blue in Figure 3d) gives a rich IR spectrum in this wavelength range with sharp and well-resolved features. The two IR spectra do not match perfectly, but are qualitatively very similar, especially in the 700–900 cm^–1^ region. The cosine similarity between the spectra is 0.974, indicating that the spectra are relatively similar. Note that the abundance of the trisaccharide in CSF is very low (<5 nM), which again likely explains some of the intensity deviations and additional noise that is observed.

Finally, we selected three tetrasaccharides: d-tetraglucoside (Glc(α1→6)Glc(α1→4)Glc(α1→4)Glc), and d-maltotetraose (Glc(α1→4)Glc(α1→4)Glc(α1→4)Glc), which differ in their first glycosidic linkage, and d-Glc-Glc-Glc-Man (Glc(α1→2)Glc(α1→3)Glc(α1→3)Man), which has both different glycosidic linkages and a different monosaccharide substituent. We observed that the tetrasaccharides, in contrast to smaller (oligo)saccharides, fragment via several pathways rather than via simple Na^+^ loss (see Figure S6), probably due to the stronger binding of Na^+^ to larger saccharides. For the tetrasaccharides, it is therefore possible to record Na^+^ adduct spectra with a good signal-to-noise ratio (in other words, record a fragmentation yield spectrum rather than a precursor depletion spectrum). Indeed, comparing the IR spectra of the Na^+^ and Cs^+^-adducts of the three tetrasaccharides (see Figure S7) shows that the two adducts give a similar level of diagnostic value, although more bands appear to be above the threshold for dissociation for the Cs^+^-adducts due to their lower dissociation threshold. As our LC–MS method showed a better sensitivity for Na^+^-adduct ions than NH_4_^+^-adduct ions, we focused here on the [M+Na]^+^ ions of the tetrasaccharides.

Figure 3e compares the IR spectra of the Na^+^-adducts of Glc-Glc-Glc-Man and d-tetraglucoside. No significant spectral broadening is observed compared to the trisaccharides, and the IR spectra are clearly distinct. d-Glc-Glc-Glc-Man is a biomarker for the rare disease MOGS-CDG, a congenital disorder of glycosylation (CDG) caused by mutations of the gene coding for glucosidase I, which is the enzyme responsible for the first step in the breakdown of N-glycans.^52^ We recorded an IR spectrum of the *m/z* 689 ion detected in the HILIC-MS analysis of urine of a MOGS-CDG patient. Figure 3e compares this IR spectrum to the reference IR spectrum of [d-Glc-Glc-Glc-Man+NH_4_]^+^, showing a very close match. The assignment is confirmed by the cosine similarity score.

d-tetraglucoside is a degradation product of branched glycogen and therefore a biomarker for a range of clinical conditions related to accumulation or increased turnover of glycogen, such as Duchenne muscular dystrophy,^53^ glycogen storage disease type I (Von Gierke disease),^54^ II (Pompe disease),^55^ III,^56^ and VI,^57^ and pregnancy.^58^ Its discrimination from d-maltotetraose, naturally occurring in plasma, is difficult using most analytical methods. Figure 3f compares the IR spectra of [d-tetraglucoside + Na]^+^ and [d-maltotetraose + Na]^+^. In this case, the differences between the spectra are very small, consistent with the fact that the two structures differ only in a single glycosidic linkage. Nevertheless, reproducible spectral details still allow distinction of the two isomeric species, for example, the peak observed in the IR spectrum of d-maltotetraose around 925 cm^–1^ and the different shape of the peaks between 500 and 600 cm^–1^. To test whether these spectral differences would be sufficient to identify a metabolite from a body fluid sample, we recorded an IR spectrum of the *m/z* 689 ion detected in the HILIC-MS analysis of urine of a patient with Von Gierke disease. Figure 3f compares this spectrum to the reference IR spectra of [d-tetraglucoside + Na]^+^ and [d-maltotetraose + Na]^+^, showing a very close match with the former of the two, especially considering the diagnostic peak around 925 cm^–1^.

### Further Discussion

Comparing all experimental IR spectra reported in this work shows that in general, the ∼600–950 cm^–1^ range contains a set of well-resolved and isolated vibrational bands, whereas the region below 600 cm^–1^ is qualitatively more congested. To get more insight into the nature of the vibrational modes in these ranges, we performed preliminary quantum-chemical calculations (see Extended Methods section in the Supporting Information) for α-d-mannose and α-d-lactose illustrative as examples. This allows us to assign vibrational normal modes to the observed IR features. A full computational (vibrational) analysis for all systems considered is beyond the scope of this study, but these preliminary results indicate that for α-d-mannose, the region below ∼550 cm^–1^ is dominated by relatively localized, overlapping hydrogen bonded C–OH bending modes. This region is relatively congested in the computed IR spectrum (see Table S4), and these bands are likely to be strongly affected by the specific hydrogen bonding network. As the ion population likely contains both α-d-mannose and β-d-mannose, potentially with a different hydrogen bonding pattern, this offers a possible explanation for the relative congestion of this region in the experimental IR spectra. The region above ∼550 cm^–1^ is predicted to be dominated by delocalized “breathing modes” involving much of the molecular ring structure and is less congested. The predicted spectrum of α-d-lactose (see Table S3) shows a similar pattern, with C–OH bending modes predicted below ∼650 cm^–1^ and delocalized breathing modes at higher wavenumbers. Interestingly, several of the latter seem to be dominated by only one of the monosaccharide constituents (i.e., the glucose or the galactose subunit). For instance, the band just below 700 cm^–1^ in the experimental spectrum is predicted to be dominated by the glucose-moiety, which explains why this band is present in all three disaccharides considered as they all contain a glucose unit. This suggests that certain individual bands in this region may indicate the presence of specific monosaccharide units. However, an extensive computational analysis on a large set of oligosaccharides is required to verify this and will be the topic of future study.

**Figure 4:**
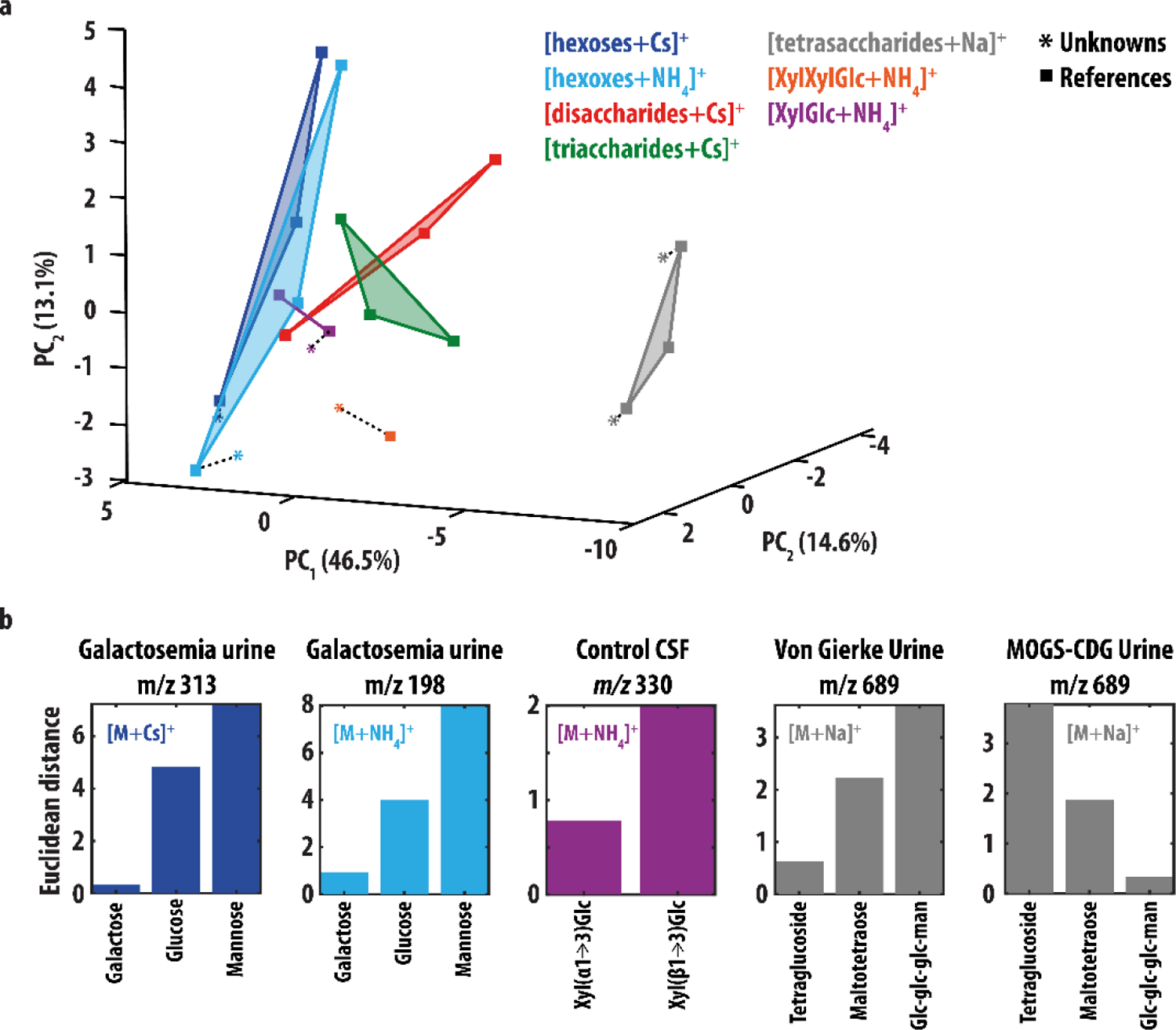
(a) Score plot resulting from PCA of the experimental IR spectra of the [M+Cs]^+^ (blue) and [M + NH_4_]^+^ (light blue) hexose ions, [M+Cs]^+^ ions of the di- (red) and trisaccharides (green), [M+Na]^+^ tetrasaccharide ions (gray), and [M+NH_4_]^+^ ions of the XylGlc and XylXylGlc saccharides (purple and orange). These reference spectra are plotted as squares. The spectra of the saccharides measured from patient samples are inlayed in the score plot as well (colored stars). A smaller distance between two points correlates with greater similarity between two spectra. The dashed lines connect the IR spectra of each unknown to it corresponding reference. (b) ED between the IR spectra of the unknowns and reference spectra with the same mass.

We used a PCA procedure to obtain information on the discriminative value of various adduct-types but it can also be applied to obtain information about the similarities and differences of oligosaccharides having different molecular mass and to provide an alternative to cosine similarity scoring for assigning structures to unknown saccharides. Therefore, we additionally performed a PCA procedure on a collection of reference IR spectra, including only the IR spectra of the Cs^+^- and NH_4_^+^-adducts for the monosaccharides and all reference spectra for the larger saccharides. This resulted in three PCs describing 72.2% of the data. The sum-of-squares of the residuals was equal to 136.8 out of a total variance of 530.2 for the data set (=25.8%). The score plot for all reference IR spectra in the three-dimensional PC_1_/PC_2_/PC_3_ space is shown in Figure 4a. Here, the distance between data points is indicative of the degree of variation between the IR spectra. As was also the case for the PCA above (Figure 2), the IR spectra recorded for the same monosaccharide (but with a different adduct ion) are grouped together. Additionally, it is seen that the tetrasaccharide spectra have a large distance to all other spectra, suggesting that their IR spectra are quite distinct. Indeed, visual comparison of the tetrasaccharide IR spectra to all other IR spectra (Figures 1 and 3) shows that they contain in general fewer observable peaks, likely due to their lower dissociation efficiency.

The PCA score plot can potentially be used for identification as well. When the IR spectrum of an unknown is plotted in the PCA score plot, it should end up closest to the reference spectrum it corresponds to as these spectra would be expected to have the highest degree of similarity. All IR spectra recorded from patient samples reported here are included in the score plot shown in Figure 4a (star symbols). Figure 4b plots their ED to the reference IR spectra of ions with the same *m/z*-value. In all cases, selection of the spectrum with the smallest distance leads to the assignment of the correct structure. This PCA procedure is thus supportive of the assignments made above using cosine similarity scoring and offers a potentially automatic and unsupervised approach.

The trisaccharide measured from control CSF as a biomarker for GLUT1DS (identified as Xyl(α1→3)Xyl(α1→3)Glc) is not included in Figure 4b as only one reference spectrum for this ion was recorded. However, Figure 4a shows that it is located relatively close (ED = 1.3) to its reference. Interestingly, the distance between the IR spectra of the trisaccharide analyte and the Xyl(α1→3)Glc disaccharide is also small (ED = 1.4, compared to ED = 3.2 for the Xyl(β1→3)Glc). These ions are very similar differing by one additional xylose moiety in the trisaccharide (also having an α linkage). This suggests that the PCA score plot could potentially provide information on the structure of an unknown saccharide even when the dataset does not contain the reference IR spectrum of its exact structure. To confirm the results of the PCA analysis, we computed the cosine similarity score between the patient IR spectra and all reference spectra. These are reported in Table S2 and are consistent with the PCA results.

## Conclusions and Outlook

This work extends the IR spectral range commonly used in IRIS studies for the differentiation of isomeric saccharides of adduct-ions to the far-IR range (300–1000 cm^–1^). While a recent review^29^ suggested that room temperature IRIS would likely only be suitable for mono- and perhaps disaccharide identification, here we show that this can readily be extended to closely isomeric tetrasaccharides and likely beyond to larger systems as well. We show that in this longer wavelength IR range, spectra of Cs^+^, Na^+^, and NH_4_^+^ complexes with saccharides have isolated, sharp, and highly diagnostic vibrations allowing their straightforward distinction. It was shown that Cs^+^-adducts are generally the preferred system for IR spectroscopy, but that NH_4_^+^ (for mono-, di- and trisaccharides) and Na^+^-adducts (for tetrasaccharides) are good alternatives to allow better compatibility with analytical LC–MS workflows. The IR spectra of larger oligosaccharides (di-, tri-, and tetrasaccharides) become increasingly congested, but still facilitate isomer distinction up to tetrasaccharides differing only by the configuration of a single glycosidic linkage. To allow for the identification of oligosaccharides directly from body fluids, we combined IRIS of NH_4_^+^ and Na^+^-adducts (which are readily formed in LC–MS compatible mobile phases) with HILIC. This combination provides a highly sensitive saccharide identification method that does not require any derivatization steps and is therefore well-suited for analytes in complex biological samples. We used this method to identify several biomarkers for IEMs, including the discovery of two novel biomarkers for GLUT1DS. This shows the added value of the developed approach as an oligosaccharide identification tool in clinical studies although we envision a wider application range in saccharide analysis, such as food chemistry.

## Supporting Information Available

Photofragmentation MS spectra, IRIS spectra, additional details on methods and data analysis, and a full description of the synthetic methods (PDF)

## Notes

The work described in this study has been carried out in accordance with The Code of Ethics of the World Medical Association (Declaration of Helsinki) for experiments involving humans. All patients (or their guardians) approved of the possible use of their anonymized left-over samples for method validation purposes, in agreement with institutional and national legislation.
