# Supplementary material for: Distinguishing Oligosaccharide Isomers Using Far-Infrared Ion Spectroscopy: Identification of Biomarkers for Inborn Errors of Metabolism
Source: Anal Chem. 2023 Jun 21;95(26):9787–96. doi: 10.1021/acs.analchem.3c00363 (PMC10323868; doi:10.1021/acs.analchem.3c00363)
Supplement: Supplementary file 1 — ac3c00363_si_001.pdf [file ac3c00363_si_001.pdf]

Supporting information:

Distinguishing oligosaccharide isomers using far-infrared ion spectroscopy - identification of biomarkers for inborn errors of metabolism

Rianne E. van Outersterp[1], Pieter C. Kooijman[1], Jona Merx[2], Udo F.H. Engelke[3], Nematollah Omidikia[4], Mei-Lan H. Tonneijck[1], Kas J. Houthuijs[1], Giel Berden[1], Tessa M.A. Peters[3,5], Dirk J. Lefeber[3,5], Michel A. A. P. Willemsen[6], Jasmin Mecinovic[7], Jeroen J. Jansen[4], Karlien L.M. Coene[3,8], Ron A. Wevers[3], Thomas J. Boltje[2], Jos Oomens[1,9], Jonathan Martens[1]*

[1]Institute for Molecules and Materials, FELIX Laboratory, Radboud University, 6525 ED Nijmegen, The Netherlands

[2]Institute for Molecules and Materials, Synthetic Organic Chemistry, Radboud University, 6525 AJ Nijmegen, The Netherlands

[3]Department of Laboratory Medicine, Translational Metabolic Laboratory, Radboud University Medical Centre, 6525 GA Nijmegen, the Netherlands

[4]Department of Analytical Chemistry and Chemometrics, Institute for Molecules and Materials, Radboud University, 6525 AJ Nijmegen, The Netherlands

[5]Department of Neurology, Donders Institute for Brain, Cognition and Behaviour, Radboud University Medical Centre, 6500 HB, Nijmegen, The Netherlands

[6]Amalia Children's Hospital, Department of Pediatric Neurology & Donders Institute for Brain, Cognition and Behaviour, Radboud University Medical Centre, 6500 HB, Nijmegen, The Netherlands

[7]University of Southern Denmark, Department of Physics, Chemistry and Pharmacy, 5230 Odense, Denmark

[8]Department of Clinical Chemistry and Hematology, Elisabeth-TweeSteden Hospital, Tilburg, The Netherlands

[9]van't Hoff Institute for Molecular Sciences, University of Amsterdam, 1098XH Amsterdam, The Netherlands

**corresponding author*

E-mail: jonathan.martens@ru.nl

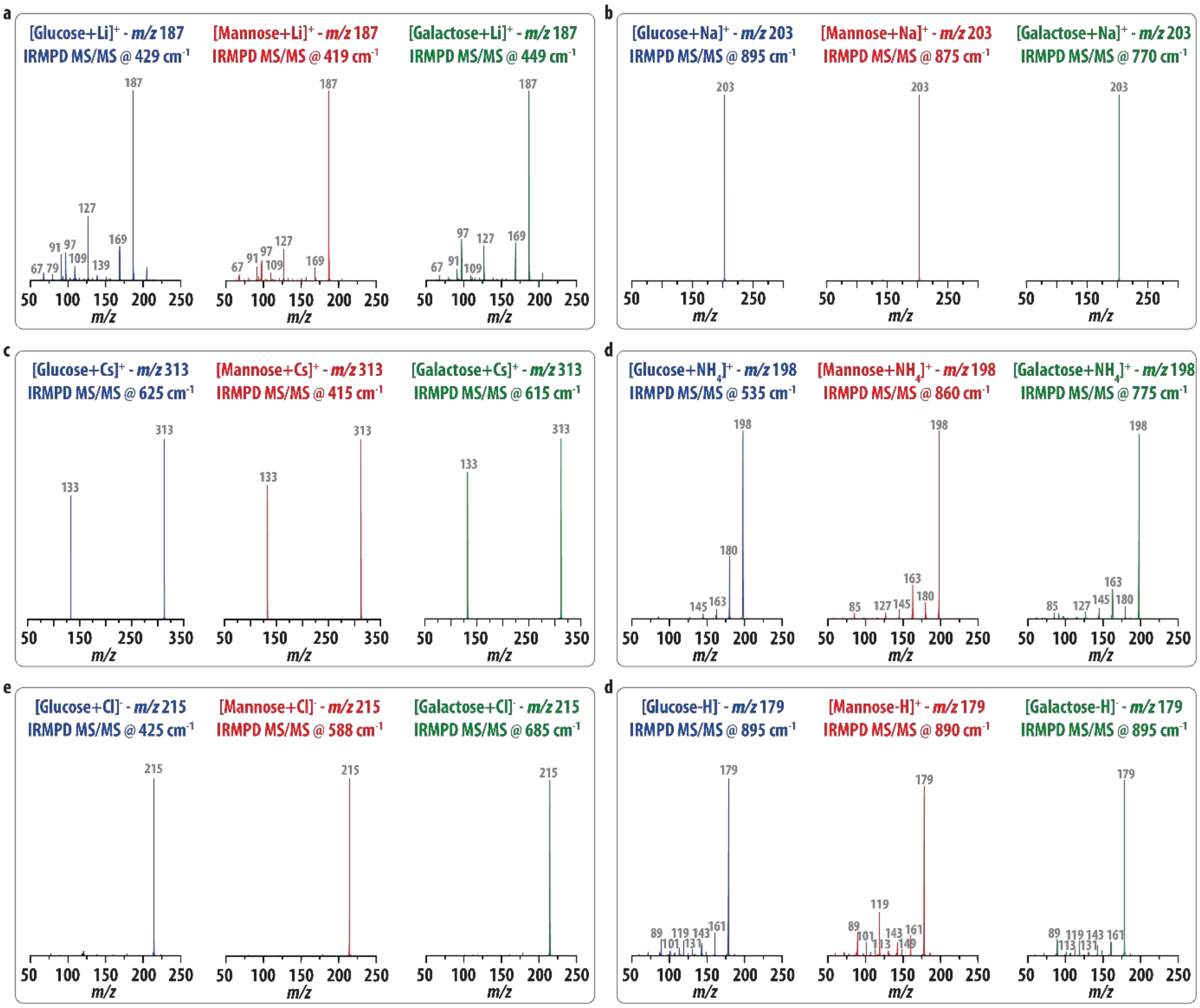

Figure S1 | *IRMPD MS/MS spectra of the (a) [M+Li]$^+$, (b) [M+Na]$^+$, (c) [M+Cs]$^+$, (d) [M+NH$_4$]$^+$, (e) [M+Cl]$^-$ and (f) [M-H]$^-$ ions of* D*-glucose (blue),* D*-mannose (red) and* D*-galactose (green). The most important peaks are labeled with their m/z value. Irradiation wavelengths and precursor ion masses are mentioned above each spectrum.*

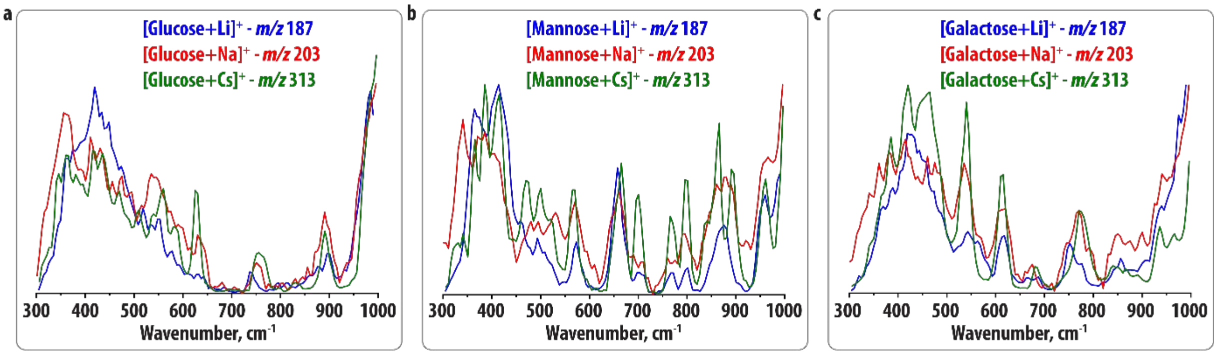

Figure S2 | *Comparison of the experimental IR spectra of the [M+Li]$^+$ (blue), [M+Na]$^+$ (red) and [M+Cs]$^+$ (green) adduct ions of (a)* D*-glucose, (b)* D*-mannose and (c)* D*-galactose*

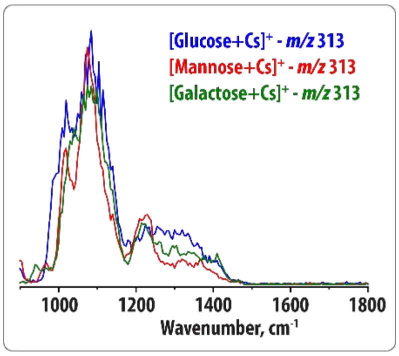

***Figure S3 |** Comparison of the experimental IR spectra of the [M+Cs]⁺ ions of D-glucose (blue), D-mannose (red) and D-galactose (green) in the fingerprint range*

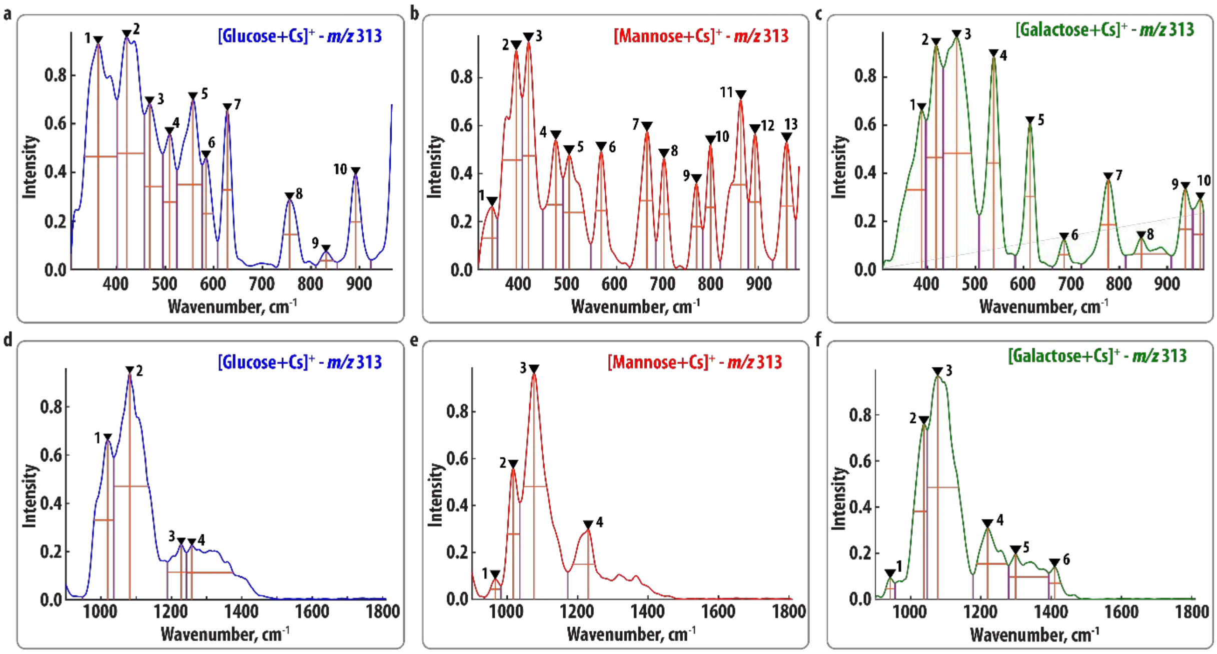

***Figure S4 |** Results of the application of a picking algorithm implemented in Matlab (see method section) to the experimental IR spectra of the [M+Cs]⁺ ions of D-glucose (blue), D-mannose (red) and D-galactose (green) in the far-IR (panel a-c, 310-995 cm^{-1}) and fingerprint (panel d-f, 900-1845 cm^{-1}) range. The position of each peak, its full width at half maximum (FWHM) and height is indicated in the figure and listed in Table S1.*

Table S1 | *Peak position, full-width at half maximum (FWHM) and height of the peaks found by a picking algorithm implemented in Matlab (see method section in the main text) in the experimental IR spectra of the [M+Cs]⁺ ions of D-glucose (blue), D-mannose (red) and D-galactose (green) in the far-IR (310-995 cm⁻¹) and fingerprint (900-1845 cm⁻¹) range.*

IR spectrum	Peak	Position (cm^{-1})	FWHM	Height
D-Glucose – far IR	1	362	67.2369	0.9307
	2	421	56.0000	0.9563
	3	468	38.0000	0.6823
	4	509	29.0000	0.5564
	5	557	52.0000	0.7001
	6	584	22.6463	0.4597
	7	628	20.6544	0.6560
	8	756	31.7961	0.2873
	9	831	29.1086	0.0735
	10	892	24.3378	0.3923
D-Mannose – far IR	1	336	14.7865	0.2615
	2	387	12.1958	0.9136
	3	413	70.4822	0.9476
	4	471	22.5603	0.5396
	5	499	13.5876	0.4767
	6	567	19.2483	0.4916
	7	664	26.8526	0.5746
	8	700	16.8282	0.4627
	9	769	14.0319	0.3579
	10	799	19.2198	0.5188
	11	863	26.6420	0.7065
	12	893	15.0077	0.5640
	13	960	19.5977	0.5302
D-Galactose – far IR	1	386	39.7460	0.6972
	2	419	38.0000	0.9826
	3	459	55.5392	0.9637
	4	539	26.8362	0.8402
	5	613	23.6461	0.5384
	6	683	22.6031	0.1108
	7	776	34.5542	0.3884
	8	844	74.2698	0.1230
	9	937	28.5776	0.2912
	10	966	19.0000	0.2774
d-Glucose – fingerprint	1	1019	55.8106	0.6612
	2	1082	99.3618	0.9404
	3	1228	54.0000	0.2286
	4	1258	132.2666	0.2253
d-Mannose – fingerprint	1	966	30.4995	0.0853
	2	1017	35.5113	0.5570
	3	1076	66.0083	0.9616
	4	1230	62.9990	0.2977
d-Galactose – fingerprint	1	942	26.8188	0.0922
	2	1038	38.9887	0.7610
	3	1077	89.6894	0.9703
	4	1219	86.5107	0.3087
	5	1299	114.0000	0.1951
	6	1410	34.7855	0.1392

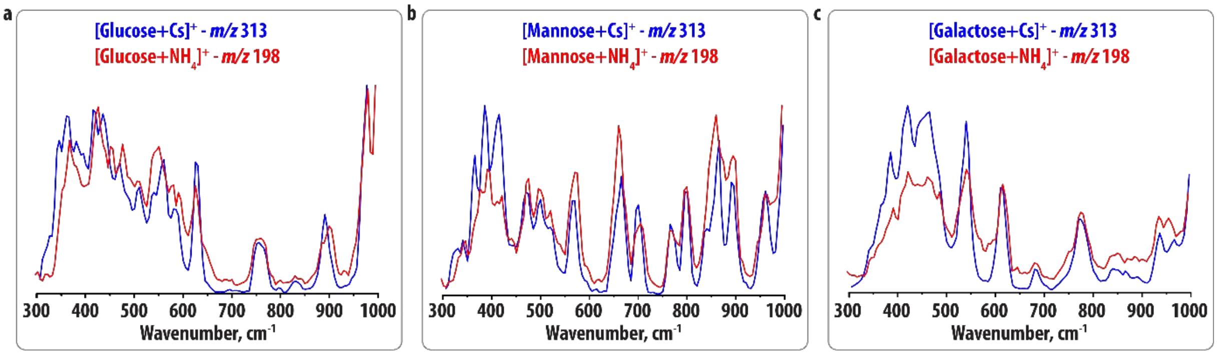

Figure S5 | *Comparison of the experimental IR spectra of the [M+Cs]$^+$ (blue) and [M+NH$_4$]$^+$ adduct (red) ions of (a) D-glucose, (b) D-mannose and (c) D-galactose*

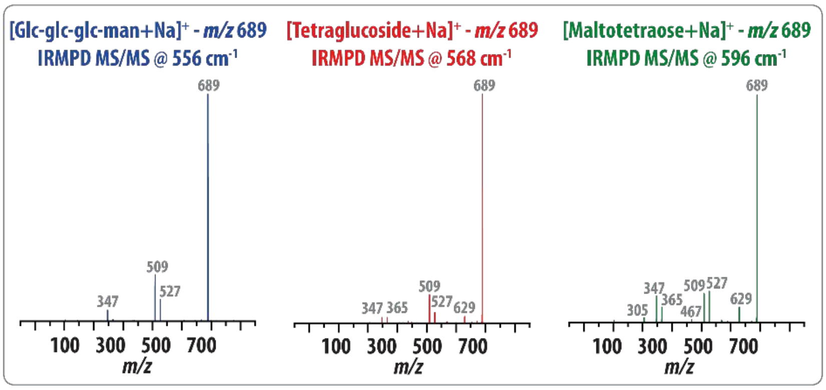

Figure S6 | *IRMPD MS/MS spectra of the [M+Na]$^+$ ions of D-Glc-glc-glc-man (blue), D-tetraglucoside (red) and D-maltotetraose (green). The most important peaks are labeled with their m/z value. Irradiation wavelengths and precursor ion masses are mentioned above each spectrum.*

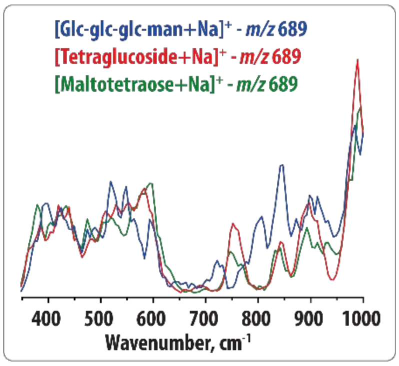

Figure S7 | *Comparison of the experimental IR spectra of the [M+Cs]$^+$ ions of D-Glc-glc-glc-man (blue), D-tetraglucoside (red) and D-maltotetraose (green).*

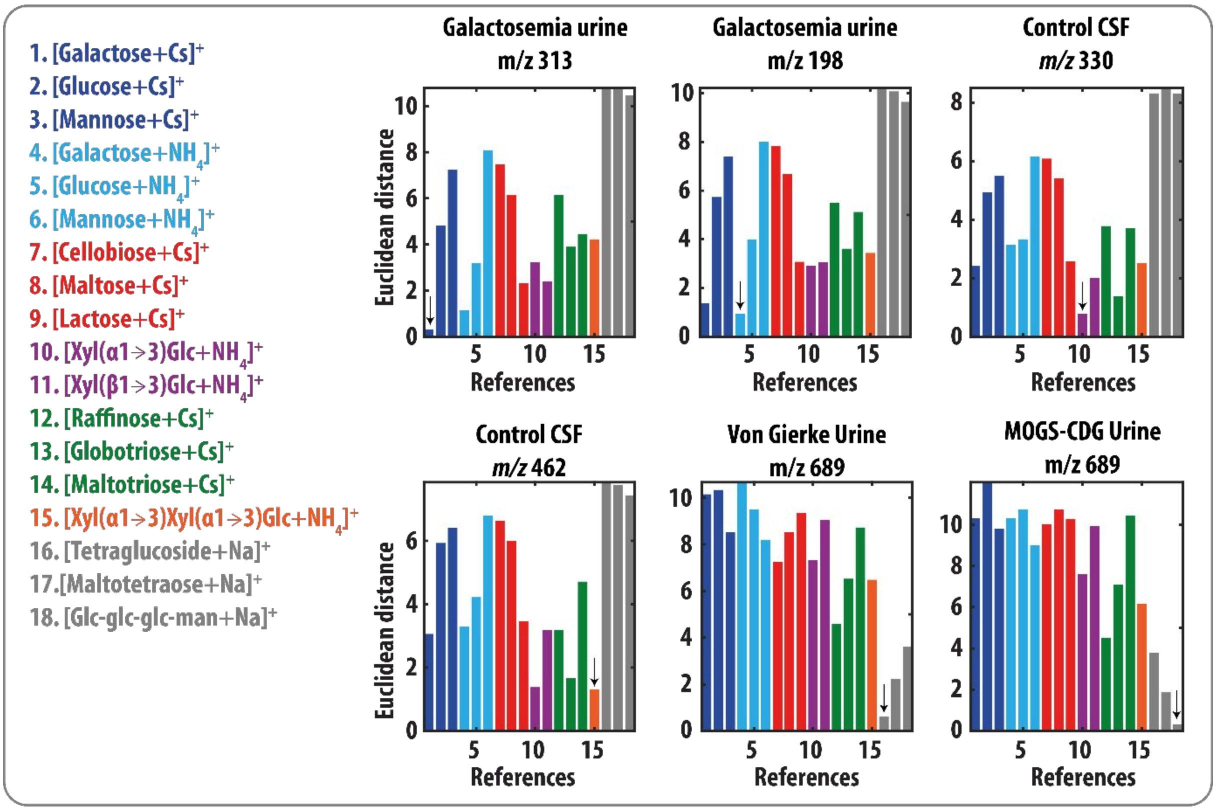

Figure S8 | *Euclidean distance between the IR spectra of the unknowns and the IR spectra of references in the PCA score plot shown in Figure 4a.*

Table S2 | *Cosine similarity scores for each of the IR spectra measured from patient samples and all reported reference spectra. The highest score (in all cases the correct spectral match) for each patient ion is highlighted.*

**Reference**	**Galactosemia urine *m/z* 313**	**Galactosemia urine *m/z* 198**	**Control CSF *m/z* 330**	**Control CSF *m/z* 462**	**Von Gierke Urine *m/z* 689**	**MOGS-CDG Urine *m/z* 689**
[Galactose+Cs]+	**0.9975**	0.9370	0.8606	0.7334	0.3343	0.3613
[Glucose+Cs]+	0.8204	0.8338	0.8552	0.7330	0.6078	0.5276
[Mannose+Cs]+	0.7282	0.7633	0.7935	0.7173	0.4724	0.4731
[Galactose+NH_4]+	0.9500	**0.9957**	0.9052	0.8772	0.5105	0.5571
[Glucose+NH_4]+	0.8772	0.9138	0.8987	0.8399	0.6122	0.5605
[Mannose+NH_4]+	0.6416	0.7506	0.7965	0.8001	0.6260	0.6168
[Cellobiose+Cs]+	0.5664	0.6518	0.6752	0.6706	0.7538	0.6388
[Maltose+Cs]+	0.6123	0.6853	0.7029	0.6730	0.7554	0.6692
[Lactose+Cs]+	0.8375	0.8412	0.8375	0.7581	0.5789	0.5079
[Xyl(α1→3)Glc +NH_4]+	0.8229	0.8765	**0.9880**	0.8634	0.5830	0.6451
[Xyl(β1→3)Glc +NH_4]+	0.7730	0.8575	0.8530	0.8149	0.7305	0.7137
[Raffinose+Cs]+	0.6562	0.7880	0.7976	0.7477	0.8157	0.8438
[Globotriose+Cs]+	0.7374	0.8394	0.8197	0.7903	0.7616	0.7729
[Maltotriose+Cs]+	0.7593	0.8264	0.8586	0.7731	0.6895	0.6603
[Xyl(α1→3)Xyl(α1→3) Glc +NH4]+	0.7318	0.8750	0.8822	**0.9711**	0.6339	0.7216
[Tetraglucoside+Na]+	0.2892	0.4588	0.4894	0.5215	**0.9837**	0.8308
[Maltotetraose+Na]+	0.3402	0.5420	0.5656	0.6223	0.9301	0.9131
[Glc-glc-glc-man+Na]+	0.3209	0.4969	0.5182	0.5559	0.8366	**0.9583**

Table S3 | Computed IR frequencies for α-D-Lactose in the far-IR range. The frequencies are scaled by 0.975.

Frequency (cm^{-1})	Intensity
310.739715	7.1732
332.76399	90.7672
351.76635	6.8629
383.4280125	3.2778
396.0651825	21.7799
402.806235	101.8971
407.345445	60.402
427.35459	2.7573
432.677115	75.9205
441.746175	31.5897
451.0044825	59.3804
473.5948425	19.0494
481.5260775	16.114
520.412685	6.5782
531.1873125	87.5332
560.56728	139.0864
589.158765	22.9324
602.4210075	33.9551
619.7843925	86.7158
629.1264525	15.5253
723.0140775	18.398
730.5207975	13.6715
777.3870975	49.559
782.3311275	18.8255
829.56237	40.9843
829.963095	4.7438
864.653595	20.9267
882.7122525	12.8404
914.9623275	2.7233
922.1180475	6.9848
949.1579175	18.9186
978.87621	9.3744
997.4367	22.7698

Table S4 | *Computed IR frequencies for α-D-Mannose in the far-IR range. The frequencies are scaled by 0.975.*

**Frequency (cm^{-1})**	**Intensity**
352.76865	*86.9479*
371.492355	*23.6935*
387.3274275	*49.744*
395.823285	*82.6924*
450.6788325	*35.5527*
468.358995	*11.6097*
486.2164125	*131.1161*
489.70779	*17.9423*
558.37938	*19.5945*
638.502345	*36.2313*
663.66261	*38.5096*
794.638455	*31.6353*
829.578165	*24.0282*
854.9356725	*17.2212*
887.6633025	*27.8626*
947.7578175	*28.0963*
986.4579075	*91.3448*

Table S5 | *Retention times of the 12 studied oligosaccharides. Details on the LC separation can be found in the method section of the main text.*

**Oligosaccharide**	**Retention time (minutes)**
D-Glucose	1.6
D-Galactose	1.6
D-Mannose	1.5
D-Cellobiose	3.2
D-Maltose	3.3
D-Lactose	3.5
D-Globotriose	5.2
D-Maltotriose	4.9
D-Raffinose	4.9
D-Tetraglucoside	6.3
D-Maltotetraose	6.0
D-Glc-glc-glc-man	5.9

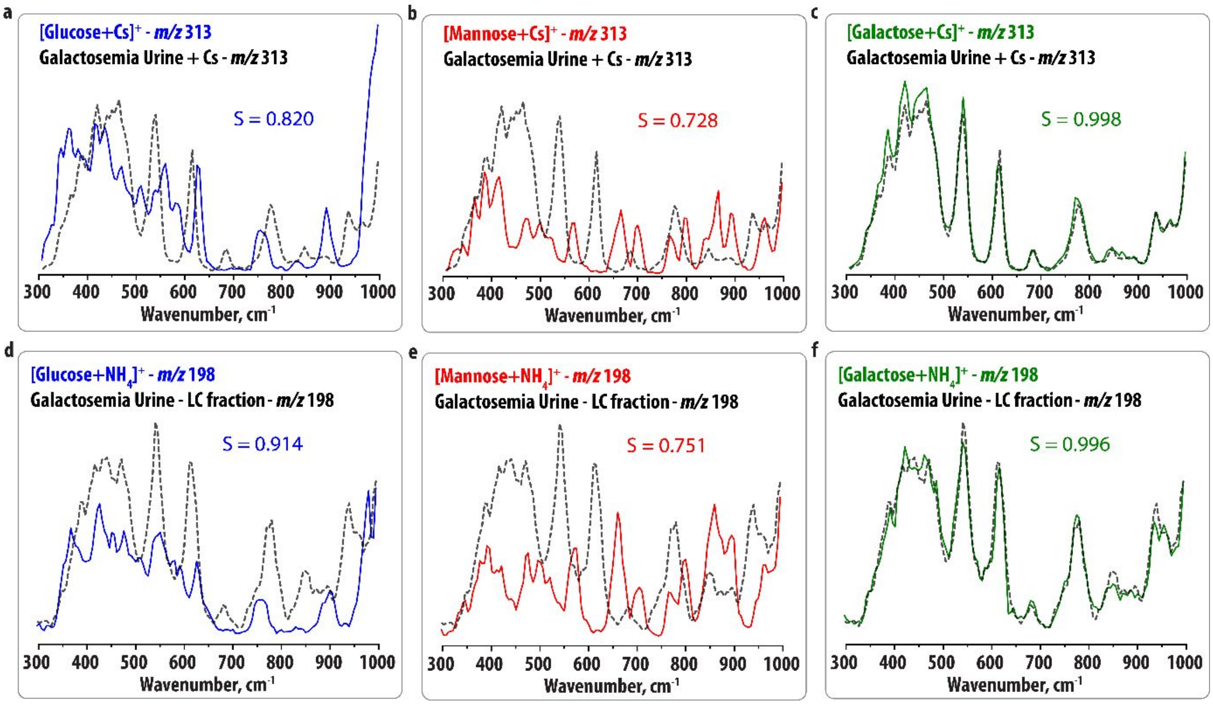

Figure S9 | *Expanded presentation of Figure 1 data.*

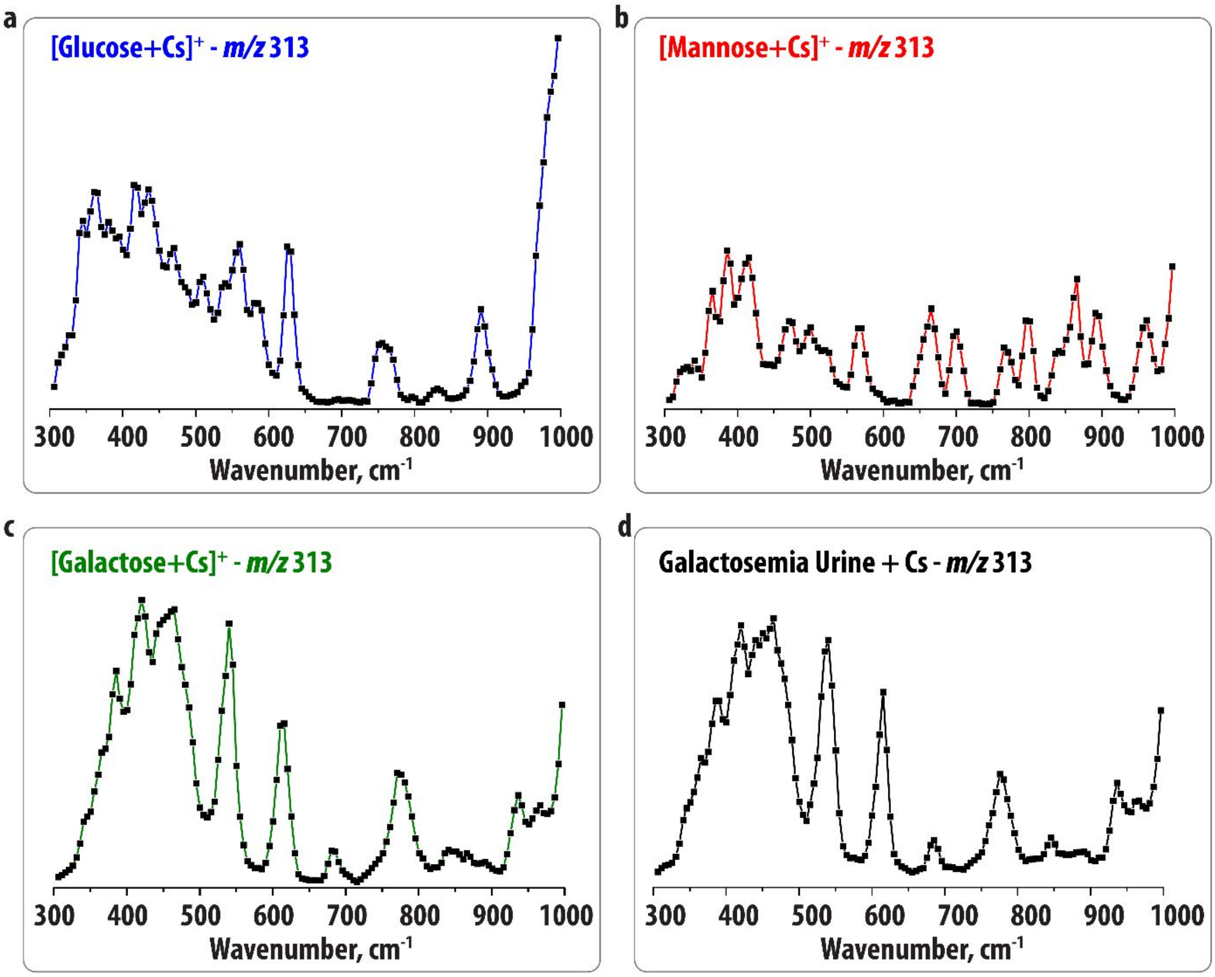

Figure S10 | *Presentation of data with raw data points and traces.*

Extended Methods

Chemicals

D-glucose, D-mannose, D-galactose, D-lactose, D-maltose, D-cellobiose, D-globotriose, D-raffinose, D-maltotriose, D-tetraglucoside and D-maltotetraose reference standards were obtained from Sigma-Aldrich Chemie (Steinheim, Germany). Purified D-glc-glc-glc-man was obtained from the urine of a MOGS-CDG patient as described in Ref. [1]. Solvents used for diluting reference standards and urine samples and mobile phase solvents were prepared with ULC/MS grade acetonitrile (ACN), water (H_2O) and methanol (MeOH) obtained from Biosolve (Valkenswaard, The Netherlands). Ammonium hydroxide (NH_4OH) was obtained from VWR International (Fontenay-sous-Bois, France). Cesium chloride (CsCl), sodium chloride (NaCl) and lithium chloride (LiCl) were obtained from Sigma-Aldrich Chemie (Steinheim, Germany). LC-MS grade MeOH, ethanol (EtOH), H_2O, and formic acid (HCOOH) used during the preparation of the CSF samples were obtained from Sigma-Aldrich (Steinheim, Germany). Reference standards for Xyl(α1→3)-Glc, Xyl(β1→3)-Glc and Xyl(α1→3)Xyl(α1→3)Glc were synthesized in-house. Synthetic procedures are found below.

Sample preparation

Commercially obtained reference compounds were analysed as ~µM solutions in 50:50 [v/v] MeOH:H_2O with 10-100 µM CsCl, LiCl or NaCl or 0.1% NH_4OH. Synthetic standards were analysed as ~µM solutions in 0.1% NH_4OH in 80:20 [v/v] MeOH:H_2O. The urine samples (stored at -80 °C) were diluted 5-10 times in ACN and centrifuged at 18600 g for 20 minutes at 4 °C. The supernatant was analysed directly with LC-MS or spiked with 100 µM CsCl for direct infusion experiments. The control CSF sample (stored at -80 °C) was thawed at 4 °C and 400 µl of ice-cold MeOH/EtOH 50:50 [v/v] was added to 100 µl of sample. The resulting sample was mixed for 15 s with a vortex mixer and incubated for 20 min at 4 °C. Subsequently, the sample was centrifuged for 15 min at 4 °C (18600 g) and the supernatant (350 µl) was dried in a centrifugal evaporator. The sample was reconstituted in 100 µl of H_2O, mixed with a vortex mixer (15 s) and centrifuged (18600 g) at room temperature. The supernatant (90 µl) was diluted with ACN and NH_4OH to a final composition of 0.1% NH_4OH in 80:20 [v/v] ACN:H_2O and analysed with LC-MS. Body fluid quantification of D-glucose and D-galactose was performed with minor modification on the method described by Jansen et al.[2]

HILIC separations

LC separations were performed with a Bruker Elute UHPLC system coupled to a quadrupole ion trap mass spectrometer (Bruker, AmaZon Speed). A Waters Acquity BEH amide column (100 x 2.1 mm i.d., 1.8 mm particles, 100 Å pore size) was used for all separations. For the analysis of the galactosemia urine sample, the column was held at a temperature of 30 °C and the mobile phase consisted of 0.1% NH_4OH in 95:5 ACN:H_2O (mobile phase A) and 0.1% NH_4OH 50:50 ACN:H_2O (mobile phase B). After an initial time of 1 minute at 100% A, a gradient was run to 100% B in 10 minutes. After a hold of 2 minutes at 100% B, the column was re-equilibrated for 14 minutes at 100% A. An injection volume of 5 µl and a flow rate of 0.3 ml/min was used. For the analysis of the GLUT1DS CSF sample, the column was held at a temperature of 35 °C and the mobile phase consisted of 0.1% NH_4OH in 80:20 ACN:H_2O (mobile phase A) and 0.1% NH_4OH 30:70 ACN:H_2O (mobile phase B). A gradient was run to 100% B in 7 minutes and the column was re-equilibrated for 16 minutes at 100% A. An injection-volume of 2–20 µl and a flow rate of 0.2 ml/min was used. The MOGS-CDG and Von Gierke disease samples were analysed using a flow rate of 0.6 ml/min and a mobile phase consisting of 0.1% NH_4OH in 80:20 ACN:H_2O (mobile phase A) and 0.1% NH_4OH 80:20 MeOH:H_2O (mobile phase B). After an initial time of 1 minute at 100% A, a gradient was run to 50% A in 8 minutes, followed by a gradient to 11.1% A in 2 minutes. After a hold of 1 minute at 11.1% A, the column was re-equilibrated for 4 minutes at 100% A. An injection volume of 10 µl and a flow rate was used and the column was held at a temperature of 55 °C. Retention times of all reference standards reported in Table S5 were also measured using this separation method. Here, an injection volume of 5 µl was used.

Infrared Ion Spectroscopy

Solutions of reference standards and the diluted urine sample were introduced at 180 µL h^{-1} flow rates to the electrospray source using a syringe pump. Fractions of LC eluent were collected using a two-position six-port switching valve controlled by the quadrupole ion trap and an 80 µL sample loop. In this set-up, the LC-eluent is diverted to waste by the switching valve until the peak of interest elutes. At that time, the valve is switched, storing the fraction of eluent in the sample loop. The valve switches back after the peak is eluted and the fraction is slowly infused by the syringe pump (120-180 µL h^{-1}).

Ions of interest were mass-isolated and stored for 180 ms in the ion trap to be irradiated with two macropulses of FELIX, which produced 300-1000 cm^{-1} radiation in the form of 5–10 µs macropulses of 10–80 mJ at 10 Hz repetition rate (bandwidth ~0.4% of the central frequency). Our IRIS experiments are based on infrared multiple-photon dissociation (IRMPD) spectroscopy: resonant absorption of IR laser light raises the internal energy of the ions and, after absorption of multiple photons, unimolecular dissociation can occur. The fractional amount of fragmentation is a measure for the degree of IR absorption at each wavelength point and an IR spectrum can be reconstructed by

relating the precursor and fragment ion intensities [IRIS intensity = ln(ΣI(precursor + fragment ions)/I(precursor ion))] as a function of wavelength.[3] When no fragment ions are observed (because the fragments are below the low mass cut-off of the ion trap) the IR spectrum is plotted as a precursor depletion spectrum [IRIS intensity = ln(I(precursor, without irradiation)/I(precursor)].[3] To obtain IR spectra, FELIX was scanned in 4 or 5 cm^{-1} steps from ~10–34 μm and a fragmentation spectrum after irradiation was obtained as an average of 6-12 scans. The IRIS intensity was linearly corrected for frequency-dependent laser pulse energy variation.[3] No smoothing was applied.

Quantum chemical calculations

In order to understand the general nature of the vibrations in the 300-1000 cm^{-1} IR region, quantum-chemical calculations were performed using the Gaussian16 software package[4] . The lowest energy conformer was selected by performing a conformational search using a workflow described previously.[5] Geometry optimizations and (harmonic) IR frequency calculations were performed using density functional theory at the B3LYP/6-31++G(d,p) level of theory. Computational frequencies were scaled by 0.975. Among the lowest-energy structures resulting from the conformational search, the conformer was assigned that showed the best match between experimental and computational spectrum.

Synthetic methods

^{1}H and ^{13}C NMR spectra were recorded on a Bruker AVANCE III (500 MHz ^{1}H, 126 MHz ^{13}C) equipped with a Bruker Prodigy BB cryoprobe or a Bruker AVANCE III (400 MHz ^{1}H, 100 MHz ^{13}C) in the solvent indicated at room temperature. Chemical shifts are reported in δ (ppm) units relative to the internal reference tetramethylsilane (Me_4Si). For ^{1}H NMR spectra, the following abbreviations are used to describe multiplicities: s (singlet), d (doublet), t (triplet), bs (broad singlet), dd (double doublet), and m (multiplet). Coupling constants are reported in Hertz (Hz) as a *J* value. Thermo Scientific™ ISQ™, high resolution MS were recorded on AccuTOF CS JMS-T100CS. All compounds were routinely checked by TLC on silica gel 60 F254 (Merck, Darmstadt, Germany); spots were visualized under UV light (254 nm) and were stained with ninhydrin, 2-4-dininitrophenylhydrazine (DNP), cerium molybdate stain or aqueous $KMnO_4$ (depending on the reaction), followed by heating. R_f values were obtained with the indicated solvent mixtures. All solvents were reagent grade and, when necessary, were purified and dried by standard methods. Organic solutions were dried over anhydrous sodium sulfate or anhydrous magnesium sulfate. The yields of the samples were calculated after drying the samples under high vacuum overnight or after lyophilization, yield corrected for residual solvents based on NMR integration. All commercially purchased reagents were used without further purification as delivered from the corresponding companies.

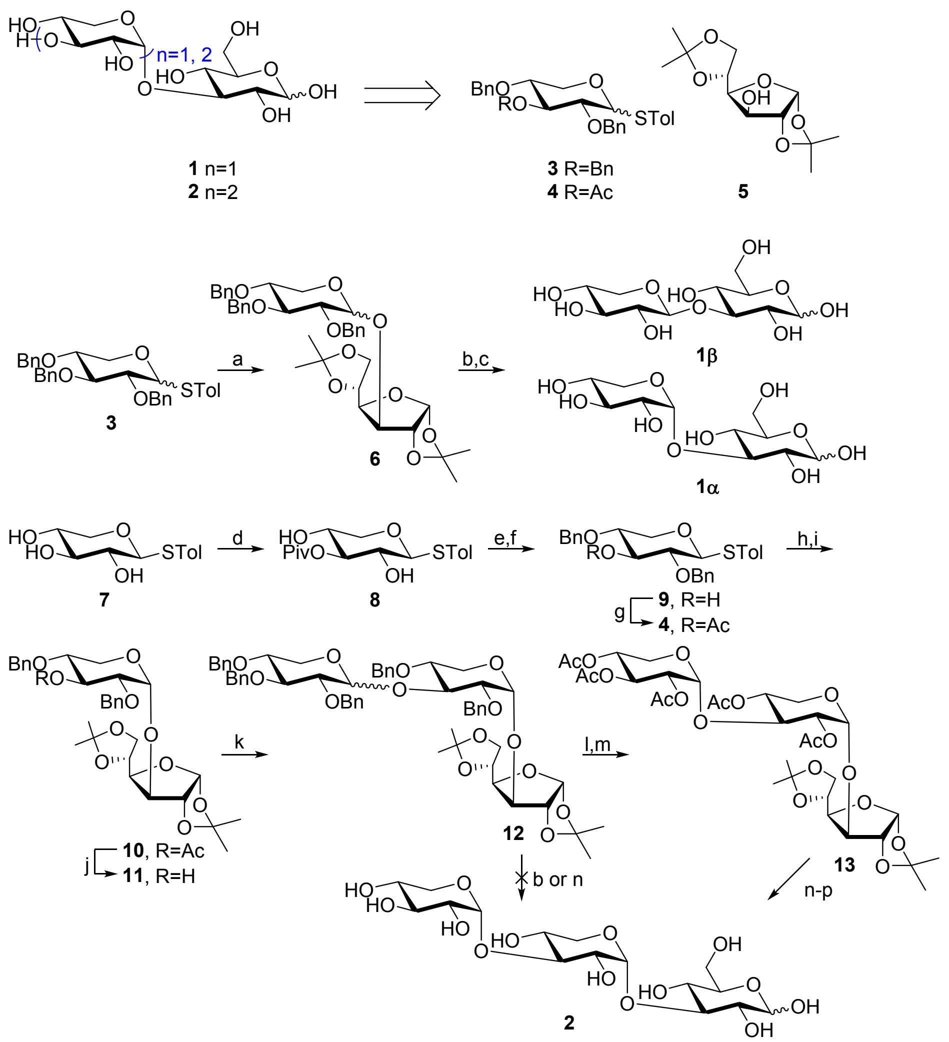

Scheme 1. Synthesis of biomarkers **1** and **2**. Reagent and conditions: **a**) **5**, NIS, TfOH, DCM:Et_2O (3:1), -50 °C, **6α** 40%, **6β** 29%; **b**) TFA:H_2O (9:1); **c**) H_2, Pd/C, MeOH, **1α** 45%, 12% **1β** over two steps; **d**) PivCl, DMAP, DCM:THF (4:1), 47%; **e**) BnBr, NaH, DMF; **f**) NaOMe, MeOH, 50 °C, 55% over two steps; **g**) Ac_2O, TEA, DMAP, DCM, 96%; **h**) **5**, NIS, TfOH, DCM:Et_2O (3:1), -50 °C, 85% (5:1, α:β); **j**) K_2CO_3, MeOH, 53%, α only; **k**) **3**, NIS, TfOH, DCM:Et_2O (3:1), -50 °C, **12α-α** 59%, **12β-α** 16%; **l**) H_2, Pd/C, MeOH; **m**) Ac_2O, DMAP, pyridine, 81% over two steps; **n**) AcOH:H_2O (8:2), 95 °c; **o**) Ac_2O, DMAP, pyridine; **p**) K_2CO_3, MeOH, 35% over three steps.

4-methylphenyl 2,3,4-tri-O-acetyl-1-thio-D-xylopyranoside (S1)

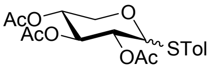

To an ice-cold solution of D-xylose (20.1 g, 134 mmol) in DCM (100 mL) and TEA (150 mL), was added DMAP (1.64 g, 13.4 mg). To this solution Ac_2O (90 mL, 950 mmol) was added dropwise. The solution was allowed to warm up to rt and was stirred overnight. The reaction was quenched with H_2O (200 mL), the layers were separated. The aqueous phase was extracted with DCM, the combined org layers were washed with 1 M HCl and sat. aq. $NaHCO_3$, dried over $MgSO_4$ and concentrated *in vacuo*. The product was obtained as a thick oil in 97% yield (41.7 g, 130 mmol) with an 1:0.13 α:β ratio. Analytical data of major α anomer given. R_f = 0.56 (1:1 EtOAc:heptane); ^{1}H NMR (400 MHz, Chloroform-*d*) δ 6.26 (d, *J* = 3.6 Hz, 1H), 5.46 (t, *J* = 9.8 Hz, 1H), 5.11 – 4.93 (m, 2H), 3.93 (dd, *J* = 11.2, 5.9 Hz, 1H), 3.78 – 3.64 (m, 1H), 2.17 (s, 3H), 2.05 (t, *J* = 1.1 Hz, 6H), 2.02 (s, 3H); ^{13}C NMR (101 MHz, Chloroform-*d*) δ 170.3, 169.91, 169.87, 169.2, 89.8, 69.5, 68.8, 60.8, 21.0, 20.9, 20.8, 20.6; HRMS (ESI$^+$): calcd for $C_{18}H_{22}NaO_7S$ $[M+Na]^+$: 405.0984, found: 405.0990.

4-methylphenyl 1-thio-D-xylopyranoside (S2)

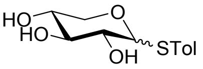

To a solution of **S1** (32.5 g, 102 mmol) in DCM (250 mL) was added thiocresol (19.0 g, 153 mmol). The reaction was cooled to 0 °C with an ice bath subsequently $BF_3.Et_2O$ (20 mL, 160 mmol) was added. The colorless solution was allowed to warm up to rt and turned deep red overnight. The reaction was quenched with sat. aq. $NaHCO_3$ (200 mL), the layers were separated. The organic layer was washed with sat. aq. $NaHCO_3$, dried over $MgSO_4$ and concentrated *in vacuo.* The crude product was dissolved in MeOH (250 mL) and NaOMe (5.4 M, 4.68 mL, 25.3 mmol) was added. Upon completion the resulting solution was concentrated *in vacuo* and recrystallized from toluene:acetone. The crystalline portion (7.06 g, 27.7mmol) contained α anomer of **7** only. The filtrate was concentrated *in vacuo* and filtered over Silica, washed with 1:1 EtOAc:heptane and concentrated *in vacuo* yielding an anomeric mixture of **7** (17.9 g, 70 mmol) with an α/β ration of 0.5:1.0 resulting in a combined yield of 80%. R_f = 0.11 (7:1 EtOAc:heptane); ^{1}H NMR (400 MHz, DMSO-d_6, α anomer) δ 7.37 – 7.28 (m, 2H), 7.21 – 6.98 (m, 2H), 5.38 – 5.27 (m, 1H), 5.22 (s, 1H), 5.05 (d, *J* = 4.2 Hz, 1H), 4.51 (d, *J* = 9.2 Hz, 1H), 3.76 (dd, *J* = 11.1, 5.1 Hz, 1H), 3.31 – 3.23 (m, 1H), 3.17 (t, *J* = 8.4 Hz, 1H), 3.10 (dd, *J* = 11.1, 9.9 Hz, 1H), 3.02 (ddd, *J* = 11.1, 8.6, 3.1 Hz, 1H), 2.27 (s, 1H); ^{13}C NMR (101 MHz, DMSO-d_6, α anomer) δ 136.5, 131.3, 130.2, 129.5, 88.1, 77.7, 71.9, 69.3, 69.1, 20.6; HRMS (ESI$^+$): calcd for $C_{12}H_{16}NaO_4S$ $[M+Na]^+$: 279.0667, found: 279.0669.

4-methylphenyl 2,3,4-tri-*O*-benzyl-1-thio-D-xylopyranoside (3)

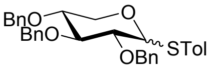

Thioglycoside **S2** (11.8 g, 46.0 mmol, anomeric mixture) was dissolved in DMF (200 mL). The solution was cooled to 0 °C and NaH (6.4 g, 161 mmol, 60 wt% dispersion in mineral oil) was added, followed by addition of BnBr (19.2 mL, 161 mmol). The solution was allowed to stir and warm up overnight to rt. The mixture was concentrated *in vacuo*, dissolved in EtOAc (200 mL) and washed 3x with H_2O (200 mL). The organic. layer was dried over $MgSO_4$ and concentrated *in vacuo* resulting in **3** obtained in 90% yield (21.9 g, 41.6 mmol). R_f = 0.84 (7:1 EtOAc:heptane); ^{1}H NMR (500 MHz, Chloroform-*d*, mixture of anomers, CH_3 of STol set to 3.0) δ 7.44 – 7.27 (m, 17H), 7.10 (dd, *J* = 8.2, 2.5 Hz, 2H), 5.47 (d, *J* = 4.7 Hz, 0.4H), 4.97 – 4.80 (m, 2.7H), 4.80 – 4.67 (m, 2.4H), 4.67 – 4.56 (m, 1.7H), 4.12 – 4.00 (m, 1H), 3.85 – 3.74 (m, 0.8H), 3.71 – 3.54 (m, 2.1H), 3.44 – 3.38 (m, 0.7H), 3.27 – 3.18 (m, 0.7H), 2.33 (s, 3H); ^{13}C NMR (126 MHz, Chloroform-*d*, mixture of anomers) δ 138.9, 138.6, 138.3, 138.2, 138.0, 137.5, 132.8, 132.4, 129.90, 129.87, 128.64, 128.59, 128.57, 128.55, 128.49, 128.3, 128.23, 128.20, 128.1, 128.04, 128.02, 127.98, 127.95, 127.9, 127.8, 88.9, 87.9, 85.6, 81.8, 79.7, 77.9, 77.8, 75.9, 75.8, 75.6, 73.7, 73.4, 72.5, 67.7, 61.1, 21.3; HRMS (ESI$^+$): calcd for $C_{33}H_{34}NaO_4S$ $[M+Na]^+$: 549.2076, found: 549.2073.

2,3,4-tri-*O*-benzyl-D-xylopyranosyl-(1-3)-1,2:5,6-di-*O*-isopropylidene-α-D-glucofuranose (6)

A solution of donor **3** (1.04 g, 1.98 mmol), acceptor **5** (619 mg, 2.38 mmol) and 3 Å molsieves in DCM:Et_2O (40 mL, 3:1) was cooled to -78 °C and kept at this temperature for 30 minutes. To this solution NIS (490 mg, 2.12 mmol) was added and stirred for an additional 15 minutes while the solution warmed up to -60 °C. TfOH (17 µL, 198 µmol) was added; the solution turned dark red at -55 °C and after 30 min the reaction was quenched with TEA (110 µL, 792 µmol). The mixture was allowed to warm up to rt and was diluted with EtOAc. The organic phase was washed with sat. aq. $NaHCO_3$ and sat. aq. NaCl, dried over $MgSO_4$ and concentrated *in vacuo*. The mixture was repeatedly purified with column chromatography (0-10 % EtOAc in cyclohexane) yielding 40% of the α anomer **6α** (531 mg, 801 µmol), 29% of β anomer of **6β** (379 mg, 572 µmol) and 6% as mixture. Analytical data for **6α**: R_f = 0.40 (3:7 EtOAc:heptane); ^{1}H NMR (500 MHz, Chloroform-*d*, α-anomer) δ 7.36 – 7.26 (m, 15H), 5.91 (d, *J* = 3.6 Hz, 1H), 5.14 (d, *J* = 3.5 Hz, 1H), 4.90 (d, *J* = 10.9 Hz, 1H), 4.85 (d, *J* = 10.9 Hz, 1H), 4.76 (d, *J* = 11.7 Hz, 2H), 4.71 (d, *J* = 11.8 Hz, 1H), 4.66 – 4.60 (m, 2H), 4.49 (ddd, *J* = 8.3, 6.2, 4.5 Hz, 1H), 4.19 (d, *J* = 2.8 Hz, 1H), 4.16 – 4.08 (m, 1H), 4.08 – 3.98 (m, 2H), 3.87 (dd, *J* = 9.6, 8.4

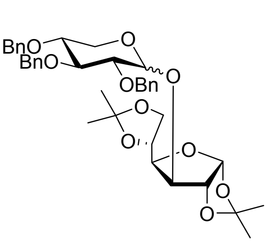

Hz, 1H), 3.75 – 3.69 (m, 1H), 3.62 – 3.50 (m, 2H), 3.46 (dd, *J* = 9.7, 3.5 Hz, 1H), 1.50 (s, 3H), 1.41 (s, 3H), 1.31 (s, 3H), 1.24 (d, *J* = 1.0 Hz, 3H); ^{13}C NMR (126 MHz, Chloroform-*d*, α anomer) δ 138.9, 138.4, 138.2, 128.7, 128.51, 128.50, 128.10, 128.08, 128.06, 127.8, 127.74, 127.67, 112.0, 109.3, 84.2, 81.4, 81.1, 80.5, 79.9, 78.1, 75.8, 74.0, 73.4, 72.4, 67.2, 61.1, 27.2, 27.0, 26.5, 25.6; HRMS (ESI$^+$): calcd for $C_{38}H_{46}NaO_{10}$ [M+Na]$^+$: 685.2989, found: 685.2968. Analytical data for **6β**: R_f = 0.38 (3:7 EtOAc:heptane); ^{1}H NMR (500 MHz, Chloroform-*d*, β-anomer) δ 7.35 – 7.26 (m, 15H), 5.72 (d, *J* = 3.8 Hz, 1H), 4.88 (d, *J* = 11.0 Hz, 1H), 4.84 (d, *J* = 11.1 Hz, 1H), 4.74 (d, *J* = 11.2 Hz, 1H), 4.71 (d, *J* = 11.7 Hz, 1H), 4.68 (d, *J* = 11.2 Hz, 1H), 4.62 (d, *J* = 11.7 Hz, 1H), 4.48 (d, *J* = 3.8 Hz, 1H), 4.41 (d, *J* = 7.6 Hz, 1H), 4.38 – 4.30 (m, 2H), 4.27 (d, *J* = 3.1 Hz, 1H), 4.05 – 4.02 (m, 2H), 3.95 (dd, *J* = 11.7, 5.0 Hz, 1H), 3.66 – 3.54 (m, 2H), 3.30 (dd, *J* = 8.8, 7.5 Hz, 1H), 3.19 (dd, *J* = 11.7, 9.5 Hz, 1H), 1.47 (s, 3H), 1.43 (s, 3H), 1.35 (s, 3H), 1.23 (s, 3H); ^{13}C NMR (126 MHz, Chloroform-*d*, β anomer) δ 136.7, 138.3, 138.2, 128.65, 128.62, 128.5, 128.08, 128.05, 128.00, 127.95, 127.8, 127.7, 112.0, 108.8, 105.2, 102.0, 83.9, 82.6, 81.7, 80.28, 80.24, 77.9, 75.7, 75.2, 73.49, 73.44, 66.2, 64.2, 26.81, 26.78, 26.2, 25.6. HRMS (ESI$^+$): calcd for $C_{38}H_{46}NaO_{10}$ [M+Na]$^+$: 685.2989, found: 685.2962.

D-xylose-(α1-3)-D-glucopyranose (1α)

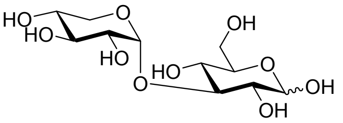

To a solution of **6α** (494 mg, 745 µmol) in DCM (1.0 mL) was added TFA:H_2O (10 mL, 9:1). The reaction was stirred at rt for 15 minutes and subsequently rapidly concentrated *in vacuo*. The residue was purified with column chromatography (80 % EtOAc in cyclohexane) resulting in a white foam (196 mg, R_f = 0.15 (4:1, EtOAc:heptane). The product was dissolved in MeOH:H_2O (32 mL, 15:1) and purged with Argon. Pd/C (36 mg, 33.9 µmol) was added, and the atmosphere was exchanged for H_2. After 16 h, the reaction mixture was filtered over Celite, concentrated *in vacuo*. The residue was purified with column chromatography (0-20%, H_2O:MeCN) and the product was lyophilized resulting in a fluffy white solid in 45% yield (105 mg, 336 µmol). R_f = 0.35 (1:4, H_2O:MeCN); ^{1}H NMR (500 MHz, D_2O, mixture of anomers, H1' s of Xylose set to 1.0) δ 5.30 – 5.25 (m, 1H), 5.17 (d, *J* = 3.8 Hz, 0.28H), 4.59 (d, *J* = 8.0 Hz, 0.82H), 3.85 – 3.74 (m, 3.4H), 3.72 – 3.45 (m, 9.25H), 3.44 – 3.37 (m, 6H), 3.31 – 3.24 (m, 0.5H); ^{13}C NMR (126 MHz, D_2O) δ 99.1, 99.0, 96.0, 92.3, 81.7, 79.7, 75.7, 73.0, 72.7, 71.67, 71.60, 71.2, 70.1, 70.0, 69.36, 69.35, 61.5, 60.5, 60.4; HRMS (ESI$^+$): calcd for $C_{11}H_{20}NaO_{10}$ [M+Na]$^+$: 335.0954, found: 335.0942.

D-xylose-(β1-3)-D-glucopyranose (1β)

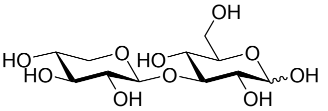

Deprotection of **6β** (365 mg, 551 µmol) analogous to **6α** resulted in a fluffy white solid of **1β** obtained in 11% yield (36 mg, 62 µmol). R_f = 0.18 (1:4, H_2O:MeCN); ^{1}H NMR (400 MHz, D_2O, α-H1 of Glucose set to 0.25) δ 5.16 (d, *J* = 3.8 Hz, 0.25H), 4.62 – 4.53 (m, 0.77H), 3.96 – 3.87 (m, 0.5H), 3.85 – 3.74 (m, 1H), 3.73 – 3.53 (m, 1.65H), 3.47 – 3.33 (m, 1.50H), 3.31 – 3.20 (m, 1H); ^{13}C NMR (101 MHz, D_2O) δ 103.6, 103.5, 95.7, 92.0, 84.3, 82.1, 75.65, 75.62, 75.59, 73.8, 73.41, 73.37, 71.3, 71.1, 69.2, 68.03, 67.97, 65.2, 60.7, 60.5; HRMS (ESI$^+$): calcd for $C_{11}H_{20}NaO_{10}$ [M+Na]$^+$: 335.0954, found: 335.0947.

4-methylphenyl 3-*O*-pivaloyl-1-thio-β-D-xylopyranoside (8)

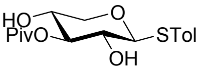

To a cooled solution of thioglycoside **S2** (3.92 g, 15.3 mmol, β only) in DCM:THF (4:1, 150 ml) were added DMAP (2.24 g, 18.4 mmol) and Piv-Cl (2.07 mL, 16.8 mmol). The solution was warmed to reflux, and kept at this temperature for 1.5 h. The reaction was quenched with the addition of sat. aq. $NaHCO_3$ (100 mL) and the layers were separated. The aqueous layer was extracted with DCM (3 x 20 mL), the combined organic layers were dried over $MgSO_4$ and concentrated *in vacuo*. The residue was purified by column chromatography (10% EtOAc in heptane) resulting in 47% yield of **8** as a white solid (2.47g, 7.26 mmol). R_f = 0.15 (3:7 EtOAc:heptane); ^{1}H NMR (500 MHz, Chloroform-*d*) δ 7.43 (d, *J* = 8.2 Hz, 2H), 7.14 (dt, *J* = 7.9, 0.8 Hz, 2H), 4.81 (t, *J* = 7.6 Hz, 1H), 4.69 (d, *J* = 7.5 Hz, 1H), 4.23 (dd, *J* = 11.9, 4.5 Hz, 1H), 3.81 – 3.70 (m, 1H), 3.60 (td, *J* = 7.5, 4.3 Hz, 1H), 3.41 (dd, *J* = 11.9, 8.3 Hz, 1H), 2.76 (d, *J* = 4.3 Hz, 1H), 2.67 (d, *J* = 5.2 Hz, 1H), 2.35 (s, 3H), 1.26 (s, 9H); ^{13}C NMR (101 MHz, Chloroform-*d*) δ 179.7 (HMBC), 133.2, 130.0, 89.7, 70.2, 68.5, 67.8, 27.2, 21.3; HRMS (ESI$^+$): calcd for $C_{17}H_{24}NaO_5S$ [M+Na]$^+$: 363.1242, found: 363.1242.

4-methylphenyl 2,4-di-*O*-benzyl-1-thio-β-D-xylopyranoside (9)

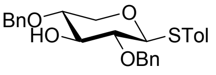

The pivaloate **8** (2.15 g, 6.32 mmol) was dissolved in DMF (30 mL), NaH (760 mg, 18.9 mmol, 60 wt% dispersion in mineral oil) was added. To this mixture BnBr (3.76 mL, 31.6 mmol) was added dropwise. The mixture was stirred overnight, quenched with the addition of sat. aq. NH_4Cl (1 mL) and concentrated *in vacuo*. The solution was taken up in EtOAc, washed with H_2O, sat. aq. $NaHCO_3$ and sat. aq. NaCl. The organic layer was dried over $MgSO_4$ and concentrated *in vacuo*. The crude mixture was dissolved in MeOH. NaOMe (1.58 mL, 8.26 mmol, 5.3 M in MeOH) was added to this solution and warmed to 50 ^{o}C overnight. The reaction was quenched with sat. aq. NH_4Cl (1 mL), diluted with H_2O and EtOAc. The layers were separated, the aqueous layer was extracted with EtOAc. The

combined organic layers were washed with sat. aq. NaCl, dried over $MgSO_4$ and concentrated *in vacuo.* The residue was purified by column chromatography (0-30% EtOAc in heptane) resulting in **9** as a white solid in 59% yield (1.63 g, 3.73 mmol) over two steps. R_f = 0.34 (30% EtOAc in heptane); ^{1}H NMR (400 MHz, Chloroform-*d*) δ 7.43 (dd, *J* = 8.2, 1.9 Hz, 4H), 7.40 – 7.27 (m, 8H), 7.15 – 7.07 (m, 2H), 4.93 (d, *J* = 10.9 Hz, 1H), 4.75 (d, *J* = 10.9 Hz, 1H), 4.69 (d, *J* = 11.8 Hz, 1H), 4.63 (d, *J* = 11.8 Hz, 1H), 4.56 (d, *J* = 9.6 Hz, 1H), 4.02 (dd, *J* = 11.4, 5.2 Hz, 1H), 3.71 (td, *J* = 8.8, 2.3 Hz, 1H), 3.49 (ddd, *J* = 10.2, 8.9, 5.1 Hz, 1H), 3.30 (dd, *J* = 9.6, 8.7 Hz, 1H), 3.18 (dd, *J* = 11.5, 10.2 Hz, 1H), 2.51 (d, *J* = 2.3 Hz, 1H), 2.34 (s, 3H); ^{13}C NMR (101 MHz, Chloroform-*d*) δ 138.3, 138.2, 138.1, 132.8, 129.9, 129.8, 128.71, 128.69, 128.3, 128.15, 128.12, 128.0, 80.6, 78.0, 77.4, 75.3, 73.2, 67.6, 21.3; HRMS (ESI$^+$): calcd for $C_{26}H_{28}NaO_4S$ [M+Na]$^+$: 459.1606, found: 459.1604.

4-methylphenyl 3-*O*-acetyl-2,4-di-*O*-benzyl-1-thio-β-D-xylopyranoside (10)

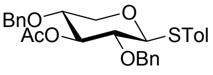

The thioglycoside **9** (1.87 g, 4.28 mmol) was dissolved in DCM (20.0 mL) and the solution was cooled to 0 °C. To this solution TEA (890 µL, 6.43 mmol), Ac_2O (525 µL, 5.57 mmol) and a catalytic amount of DMAP (52.3 mg, 0.43 mmol) were added. The solution was allowed to warm up to rt and was stirred for 3 h. The reaction mixture was quenched with H_2O, the layers were separated. The organic layer was washed with sat. aq. $NaHCO_3$ and 1 M HCl. The combined organic layers were dried over $MgSO_4$ and concentrated *in vacuo*. Purification of the residue with column chromatography resulted in **4** obtained as white solid in 96% yield (1.98 g, 4.28 mmol). R_f = 0.42 (30% EtOAc in heptane); ^{1}H NMR (400 MHz, Chloroform-*d*) δ 7.44 – 7.38 (m, 2H), 7.36 – 7.21 (m, 10H), 7.16 – 7.08 (m, 2H), 5.20 (t, *J* = 8.8 Hz, 1H), 4.85 (d, *J* = 11.0 Hz, 1H), 4.66 (d, *J* = 9.1 Hz, 1H), 4.60 – 4.48 (m, 3H), 4.06 (dd, *J* = 11.6, 5.1 Hz, 1H), 3.52 (ddd, *J* = 9.8, 8.9, 5.0 Hz, 1H), 3.39 (t, *J* = 8.9 Hz, 1H), 3.29 (dd, *J* = 11.6, 9.8 Hz, 1H), 2.34 (s, 3H), 1.93 (s, 3H); ^{13}C NMR (101 MHz, Chloroform-*d*) δ 170.0, 138.2, 138.0, 137.9, 132.8, 129.9, 129.7, 128.61, 128.55, 128.2, 128.1, 128.0, 127.9, 88.8, 78.8, 76.0, 75.3, 74.8, 72.8, 67.3, 21.3, 21.1; HRMS (ESI$^+$) calcd for $C_{28}H_{30}NaO_5S$ [M+Na]$^+$: 501.1712, found: 501.1710.

2,4-di-*O*-benzyl-β-D-xylopyranoside-(α1-3)-1,2:5,6-di-*O*-isopropylidene-α-D-glucofuranose (11)

A solution of donor **4** (920 mg, 1.92 mmol), acceptor **5** (600 mg, 2.31 mmol) and 3 Å molsieves in DCM:Et_2O (40 mL, 3:1) was

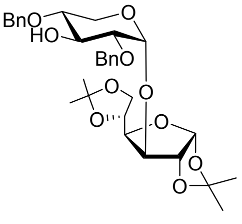

cooled to -78 °C and kept at this temperature for 30 minutes. To this solution NIS (476 mg, 2.11 mmol) was added and stirred for an additional 15 minutes while the solution warmed up to -60 °C. TfOH (17 µL, 198 µmol) was added; the solution turned dark red at -52 °C and after 30 min the reaction was quenched with TEA (107 µL, 769 µmol). The mixture was allowed to warm up to rt and was diluted with EtOAc. The organic phase was washed with sat. aq. $NaHCO_3$ and sat. aq. NaCl, dried over $MgSO_4$ and concentrated *in vacuo*. The mixture was purified with column chromatography (0-15% EtOAc in cyclohexane) yielding an inseparable mixture of anomers in a 4:1 α:β ratio (1.01 g, 1.64 mmol, R_f = 0.32 (3:7 EtOAc:heptane)). To a solution of the anomeric mixture (1.0 g, in 1.64 mmol) in MeOH (10 mL) was added solid K_2CO_3 (45 mg, 0.33 mmol). The reaction was stirred at rt for 2 h, the solution was neutralized with Amberlyst (15 hydrogen form), filtered and concentrated *in vacuo*. The residue was purified with column chromatography (0-16% EtOAc in cyclohexane) resulting in the isolation of the α anomer as a white solid in 53% yield (491 mg, 0.85 mmol). R_f = 0.27 (3:7 EtOAc:heptane); ^{1}H NMR (500 MHz, Chloroform-*d*) δ 7.39 – 7.27 (m, 10H), 5.88 (d, *J* = 3.6 Hz, 1H), 5.15 (d, *J* = 3.5 Hz, 1H), 4.87 – 4.74 (m, 2H), 4.67 – 4.60 (m, 2H), 4.59 (d, *J* = 3.6 Hz, 1H), 4.46 (ddd, *J* = 8.5, 6.2, 4.4 Hz, 1H), 4.17 (d, *J* = 2.8 Hz, 1H), 4.08 (dd, *J* = 8.6, 2.8 Hz, 1H), 4.06 – 3.96 (m, 3H), 3.75 – 3.65 (m, 1H), 3.57 – 3.44 (m, 2H), 3.33 (dd, *J* = 9.7, 3.5 Hz, 1H), 2.50 (d, *J* = 2.0 Hz, 1H), 1.49 (s, 3H), 1.42 (s, 3H), 1.31 (s, 3H), 1.24 (s, 3H); ^{13}C NMR (126 MHz, Chloroform-*d*) δ 138.3, 138.0, 128.7, 128.13, 128.10, 128.06, 127.8, 112.1, 109.3, 105.3, 97.6, 84.1, 81.4, 80.7, 79.3, 77.5, 73.6, 72.9, 72.7, 72.3, 67.3, 60.6, 27.2, 27.0, 26.4, 25.6; HRMS (ESI$^+$): calcd for $C_{31}H_{40}NaO_{10}$ [M+Na]$^+$: 595.2519, found: 595.2502.

2,3,4-tri-*O*-benzyl-D-xylopyranoside-(1-3)-2,4-di-*O*-benzyl-D-xylopyranoside-(α1-3)-1,2:5,6-di-*O*-isopropylidene-α-D-glucofuranose (12)

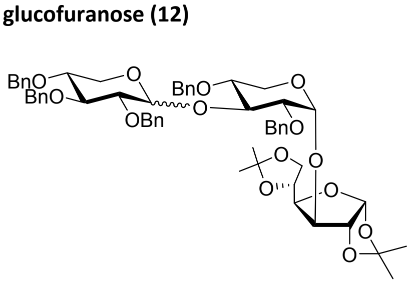

A solution of donor **3** (542 mg, 1.03 mmol), acceptor 10 (491 mg, 857 µmol) and 3 Å molsieves in DCM:Et_2O (40 mL, 3:1) was cooled to -78 °C and kept at this temperature for 30 minutes. To this solution NIS (212 mg, 0.94 mmol) was added and stirred for an additional 15 minutes while the solution warmed up to -60 °C. TfOH (7 µL, 86 µmol) was added; the solution turned dark red at -55 °C and after 30 min the reaction was quenched with TEA (120 µL, 857 µmol). The mixture was allowed to warm up to rt and was diluted with EtOAc. The organic phase was washed with sat. aq. $NaHCO_3$ and sat. aq. NaCl, dried over $MgSO_4$ and concentrated *in vacuo*. The mixture was purified with repeated column chromatography (0-15% EtOAc in heptane) resulting in the isolation of the desired α-α anomer **12α-α** as white solid in 59 % yield (497 mg, 510 µmol) and isolation of the β-α

anomer **12β-α** in 16% yield (137 mg, 140 µmol). Analytical data given of the desired α-α anomer. R_f = 0.33 (3:7 EtOAc:heptane); ^{1}H NMR (500 MHz, Chloroform-*d*) δ 7.41 – 7.14 (m, 28H), 5.92 (d, *J* = 3.6 Hz, 1H), 5.57 (d, *J* = 3.6 Hz, 1H), 5.13 (d, *J* = 3.5 Hz, 1H), 4.88 (s, 2H), 4.73 (d, *J* = 11.5 Hz, 1H), 4.69 (d, *J* = 11.6 Hz, 1H), 4.65 (d, *J* = 12.0 Hz, 1H), 4.64 – 4.54 (m, 4H), 4.48 (d, *J* = 11.7 Hz, 1H), 4.46 – 4.41 (m, 1H), 4.18 – 4.09 (m, 3H), 4.06 – 3.99 (m, 2H), 3.95 – 3.88 (m, 2H), 3.76 – 3.64 (m, 2H), 3.58 – 3.46 (m, 4H), 3.44 (dd, *J* = 9.7, 3.6 Hz, 1H), 1.49 (s, 3H), 1.40 (s, 3H), 1.31 (s, 3H), 1.16 (s, 3H); ^{13}C NMR (126 MHz, Chloroform-*d*) δ 139.1, 138.7, 138.3, 138.2, 138.0, 128.6, 128.45, 128.42, 128.40, 128.39, 128.1, 128.0, 127.91, 127.89, 127.8, 127.68, 127.65, 127.61, 127.3, 112.0, 109.2, 105.4, 98.0, 97.0, 84.1, 81.3, 80.8, 79.7, 79.4, 78.7, 78.2, 75.7, 74.2, 73.4, 73.1, 73.0, 72.8, 72.4, 66.7, 60.5, 60.3, 27.2, 27.0, 26.5, 25.4; HRMS (ESI$^+$)+ calcd for $C_{57}H_{66}NaO_{14}$ [M+Na]$^+$: 997.4350, found: 997.4307.

2,3,4-tri-*O*-acetyl-D-xylopyranoside-(α1-3)-2,4-di-*O*-acetyl-D-xylopyranoside-(α1-3)-1,2:5,6-di-*O*-isopropylidene-α-D-glucofuranose (13)

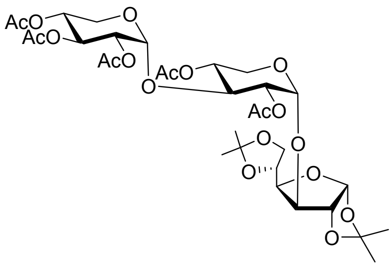

A solution of trisaccharide **12α-α** (350 mg, 359 µmol) in MeOH (10 mL) was degassed with Argon. A catalytic amount of Pd/C (40 mg, 35.9 µmol, 10wt%) was added and the atmosphere was exchanged to H_2. The reaction was stirred for 4 days obtaining full conversion (R_f = 0.15 (1:9 MeOH:DCM)) and was subsequently filtered over Celite and concentrated *in vacuo*. The residue was dissolved in pyridine (10 mL) and Ac_2O (406 µL, 4.31 mmol) was added as well as a catalytic amount of DMAP (4.4 mg, 36 µmol). The reaction was stirred overnight and subsequently concentrated *in vacuo*. The residue was taken up in EtOAc, washed with sat. aq. $CuSO_4$, sat. aq. $NaHCO_3$ and sat. aq. NaCl. The organic layer was dried over $MgSO_4$ and concentrated *in vacuo*. The resulting residue was purified with column chromatography (0-50% EtOAc in heptane) resulting in the acetylated trisaccharide **13** as a white foam in 81% yield (214 mg, 291 µmol). R_f = 0.23 (1:1 EtOAc:heptane); ^{1}H NMR (500 MHz, Chloroform-*d*) δ 5.94 (d, *J* = 3.6 Hz, 1H), 5.42 – 5.33 (m, 2H), 5.27 (d, *J* = 3.7 Hz, 1H), 5.07 – 4.94 (m, 2H), 4.86 (dd, *J* = 10.1, 3.7 Hz, 1H), 4.74 (dd, *J* = 10.3, 3.7 Hz, 1H), 4.60 (d, *J* = 3.6 Hz, 1H), 4.30 – 4.25 (m, 1H), 4.21 – 4.06 (m, 5H), 3.88 – 3.76 (m, 3H), 3.48 (t, *J* = 10.8 Hz, 1H), 2.11 (s, 3H), 2.10 (s, 3H), 2.06 (s, 3H), 2.04 (s, 3H), 2.03 (s, 3H), 1.52 (s, 3H), 1.43 – 1.40 (m, 3H), 1.35 – 1.32 (m, 6H); ^{13}C NMR (126 MHz, Chloroform-*d*) δ 170.5, 169.9, 169.8, 169.5, 112.2, 109.5, 105.2, 97.3, 95.4, 83.8, 81.5, 81.1, 72.6, 72.3, 71.7, 71.5, 71.1, 69.13, 69.11, 67.4, 58.9, 58.5, 27.1, 26.9, 26.3, 25.4, 20.84, 20.84, 20.73, 20.67, 20.6; HRMS (ESI$^+$): calcd for $C_{32}H_{46}NaO_{19}$ [M+Na]$^+$: 757.2531, found: 757.2521.

D-xylose-(α1-3)-D-xylose-(α1-3)-D-glucopyranose (2)

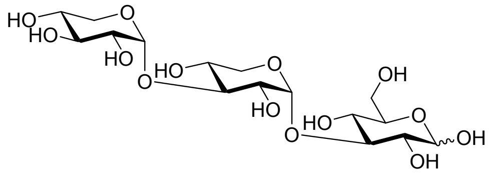

Acetylated trisaccharide **12** (200 mg, 272 µmol) was dissolved in AcOH:H_2O (100 mL, 8:2) and the resulting solution was heated to 95 °C overnight. The solution was allowed to cool to rt, the solvent was removed *in vacuo*. The residue was dissolved in pyridine (5 mL) and Ac_2O (385 µL, 4.08 mmol) was added as well as a catalytic amount of DMAP (3.3 mg, 27 µmol). The reaction was stirred overnight and subsequently concentrated *in vacuo*. The residue was taken up in EtOAc, washed with sat. aq. $CuSO_4$, sat. aq. $NaHCO_3$ and sat. aq. NaCl. The organic layer was dried over $MgSO_4$ and concentrated *in vacuo*. The resulting residue was purified with column chromatography (40-70% EtOAc in heptane) resulting in a white foam contaminated with an inseparable peracetyled glucose (R_f = 0.51 (8:2 EtOAc:heptane)). The mixture was dissolved in MeOH (5 mL) and solid K_2CO_3 (10 mg) was added. The solution was stirred for 2 h, neutralized with Amberlyst (15 hydrogen form), filtered and concentrated *in vacuo*. The mixture was purified by column chromatography (0-20% H_2O in MeCN) and subsequent gel filtration chromatography (Biorad P2 gel). The product was lyophilized to obtain **2** as a fluffy white solid in 35% yield (43 mg, 97 µmol). R_f = 0.18 (1:4 H_2O:MeCN); ^{1}H NMR (500 MHz, D_2O) δ 5.38 – 5.32 (m, 2H), 5.26 (d, *J* = 3.8 Hz, 0.4H), 4.68 (d, *J* = 8.0 Hz, 0.6H), 3.95 – 3.60 (m, 14.4H), 3.57 (dd, *J* = 9.6, 3.8 Hz, 1H), 3.49 (ddd, *J* = 9.6, 5.8, 2.3 Hz, 0.6H), 3.37 (dd, *J* = 8.9, 7.9 Hz, 0.6H); ^{13}C NMR (126 MHz, D_2O) δ 99.3, 99.1, 99.0, 96.0, 92.2, 81.9, 79.4, 79.1, 75.6, 73.0, 72.7, 71.6, 71.2, 70.11, 70.05, 70.02, 69.7, 69.4, 61.6, 61.5, 60.5, 60.4; HRMS (ESI$^+$) calcd for $C_{16}H_{28}NaO_{14}$ [M+Na]$^+$: 467.1378, found: 467.1395.

NMR Spectra

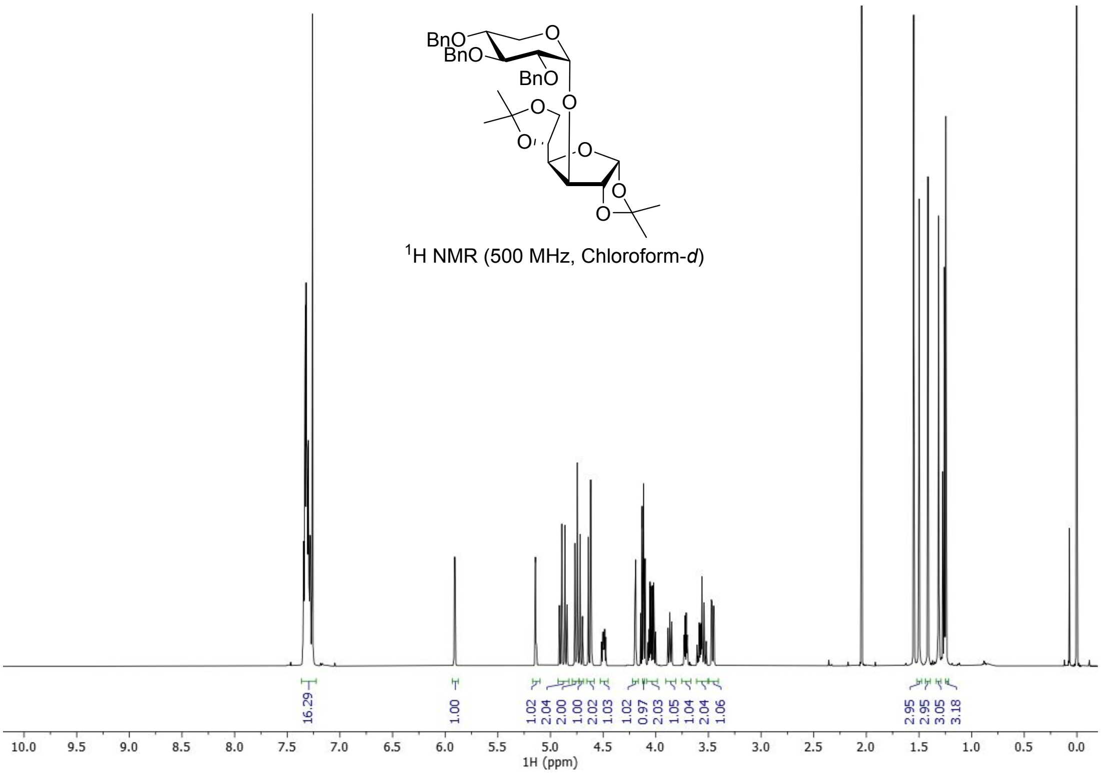

BnO
BnO
BnO
O
^{1}H NMR (500 MHz, Chloroform-*d*)
16.29
1.00
1.02
2.04
2.00
1.00
2.02
1.03
1.02
0.97
2.03
1.05
1.04
2.04
1.06
2.95
2.95
3.05
3.18
1H (ppm)

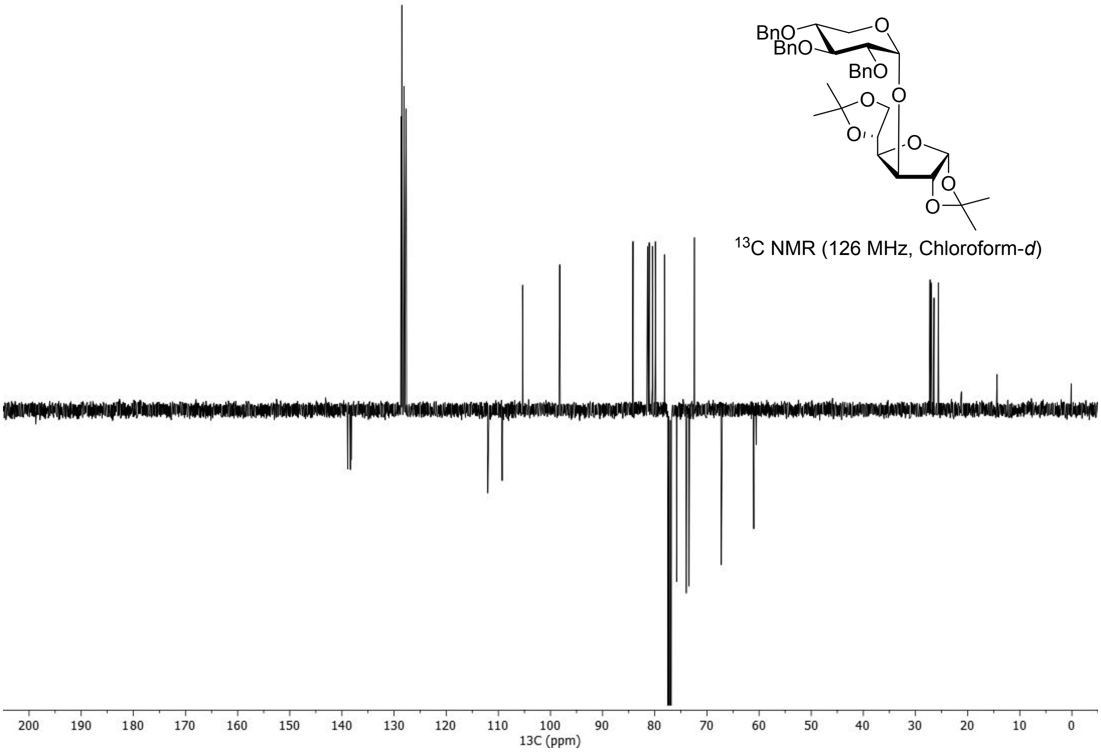

BnO
BnO
BnO
O
^{13}C NMR (126 MHz, Chloroform-*d*)
13C (ppm)

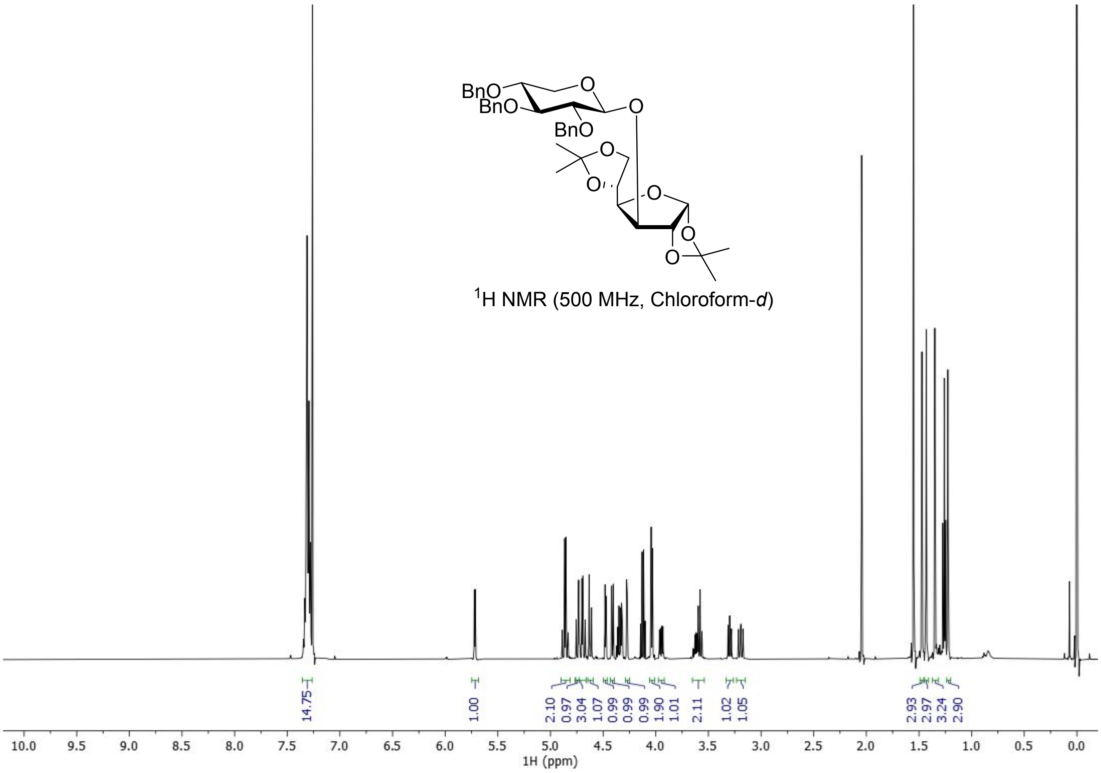
BnO
BnO
BnO
O
^{1}H NMR (500 MHz, Chloroform-*d*)
14.75
1.00
2.10
0.97
3.04
1.07
0.99
0.99
0.99
1.90
1.01
2.11
1.02
1.05
2.93
2.97
3.24
2.90
10.0 9.5 9.0 8.5 8.0 7.5 7.0 6.5 6.0 5.5 5.0 4.5 4.0 3.5 3.0 2.5 2.0 1.5 1.0 0.5 0.0
1H (ppm)

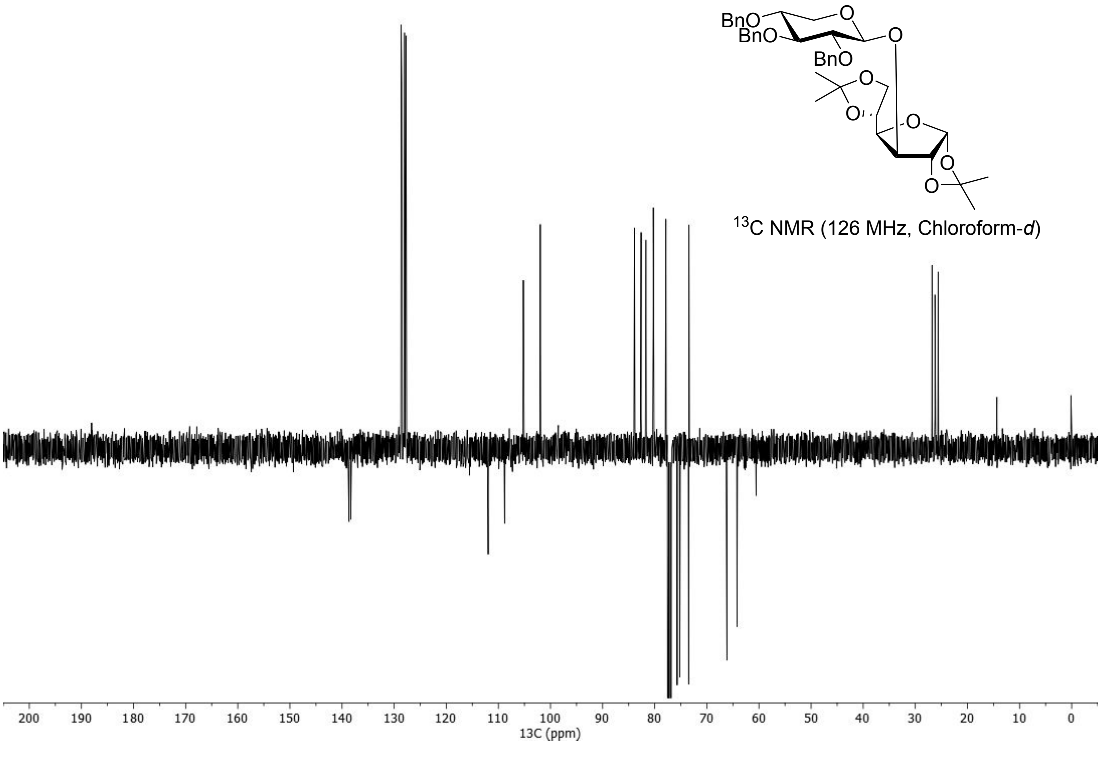
BnO
BnO
BnO
O
^{13}C NMR (126 MHz, Chloroform-*d*)
200 190 180 170 160 150 140 130 120 110 100 90 80 70 60 50 40 30 20 10 0
13C (ppm)

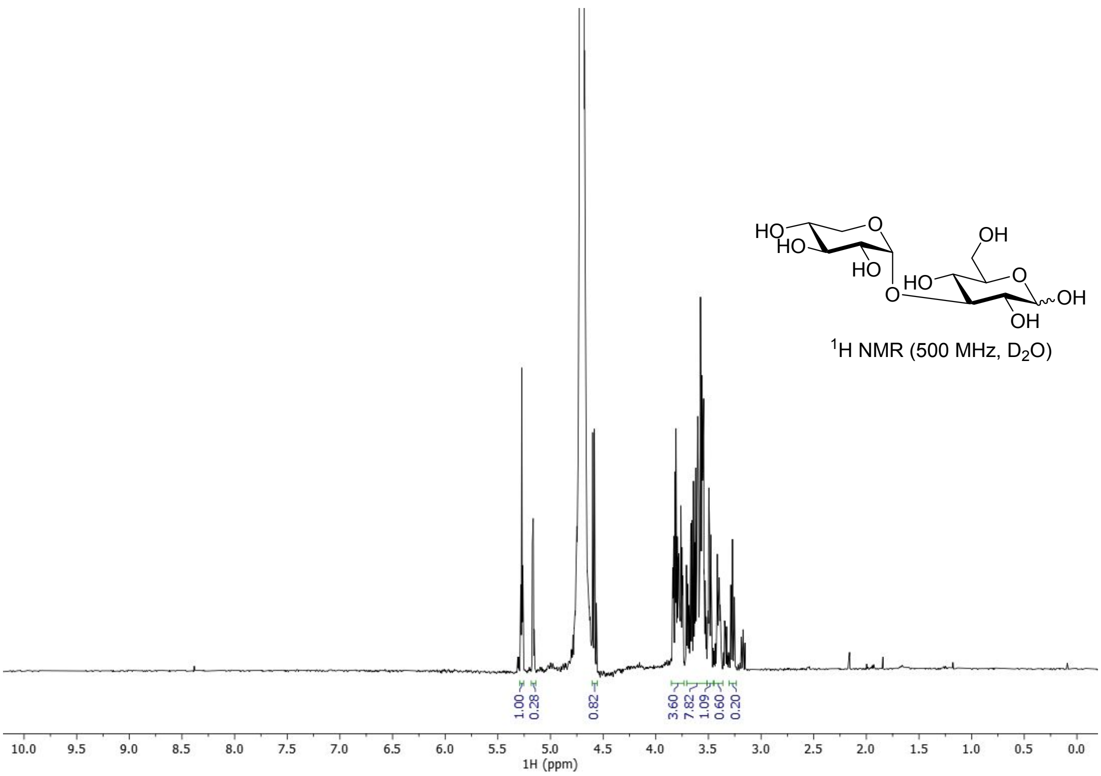
1H NMR (500 MHz, D2O)
1.00
0.28
0.82
3.60
7.82
1.09
0.60
0.20
1H (ppm)

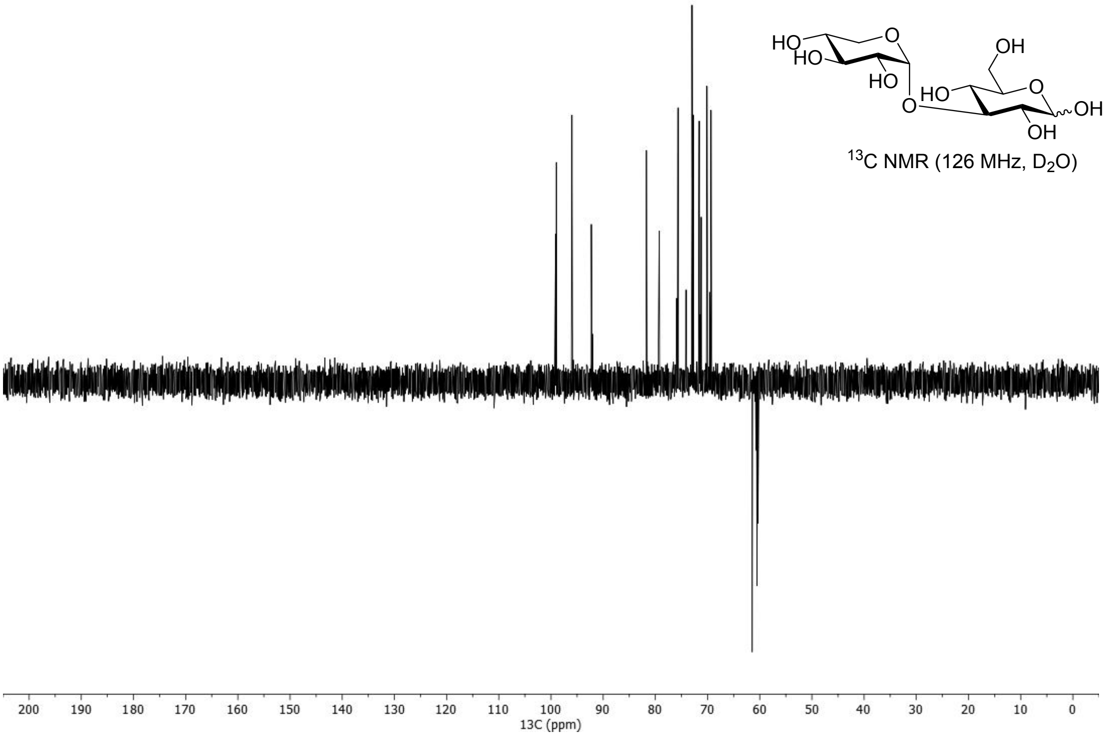
13C NMR (126 MHz, D2O)
13C (ppm)

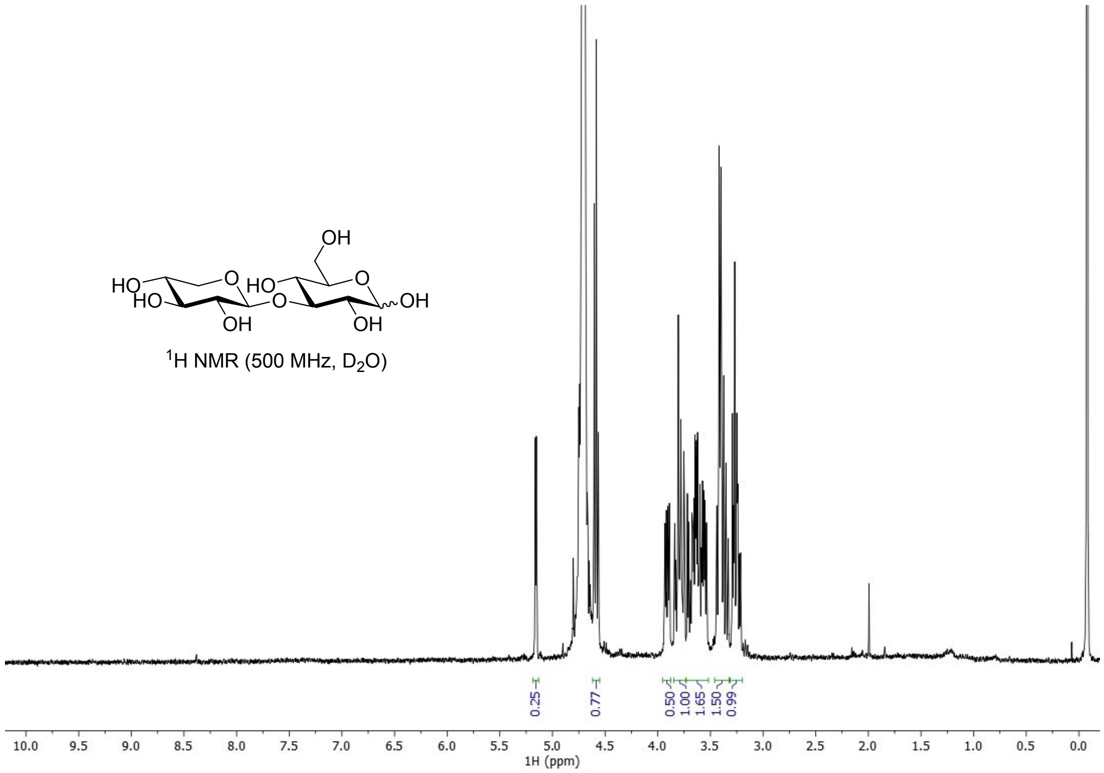
OH
HO
O
HO
HO
O
O
OH
OH
OH
OH
¹H NMR (500 MHz, D₂O)
0.25
0.77
0.50
1.00
1.65
1.50
0.99
10.0 9.5 9.0 8.5 8.0 7.5 7.0 6.5 6.0 5.5 5.0 4.5 4.0 3.5 3.0 2.5 2.0 1.5 1.0 0.5 0.0
1H (ppm)

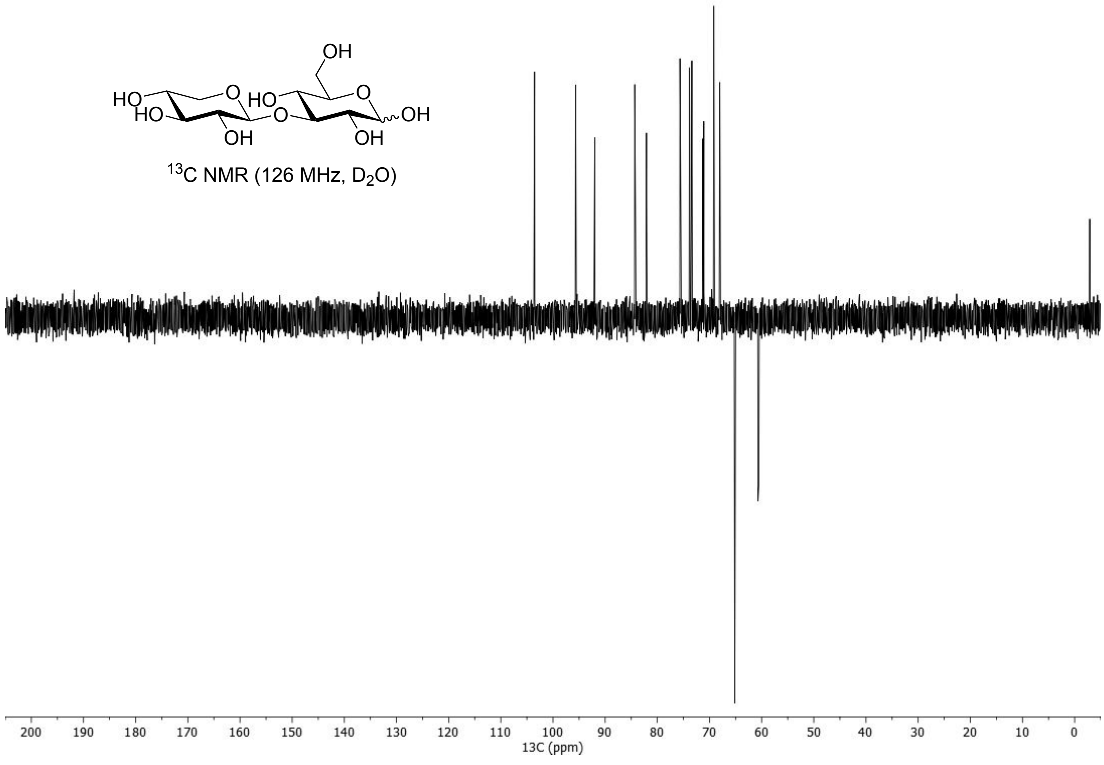
OH
HO
O
HO
HO
O
O
OH
OH
OH
OH
¹³C NMR (126 MHz, D₂O)
200 190 180 170 160 150 140 130 120 110 100 90 80 70 60 50 40 30 20 10 0
13C (ppm)

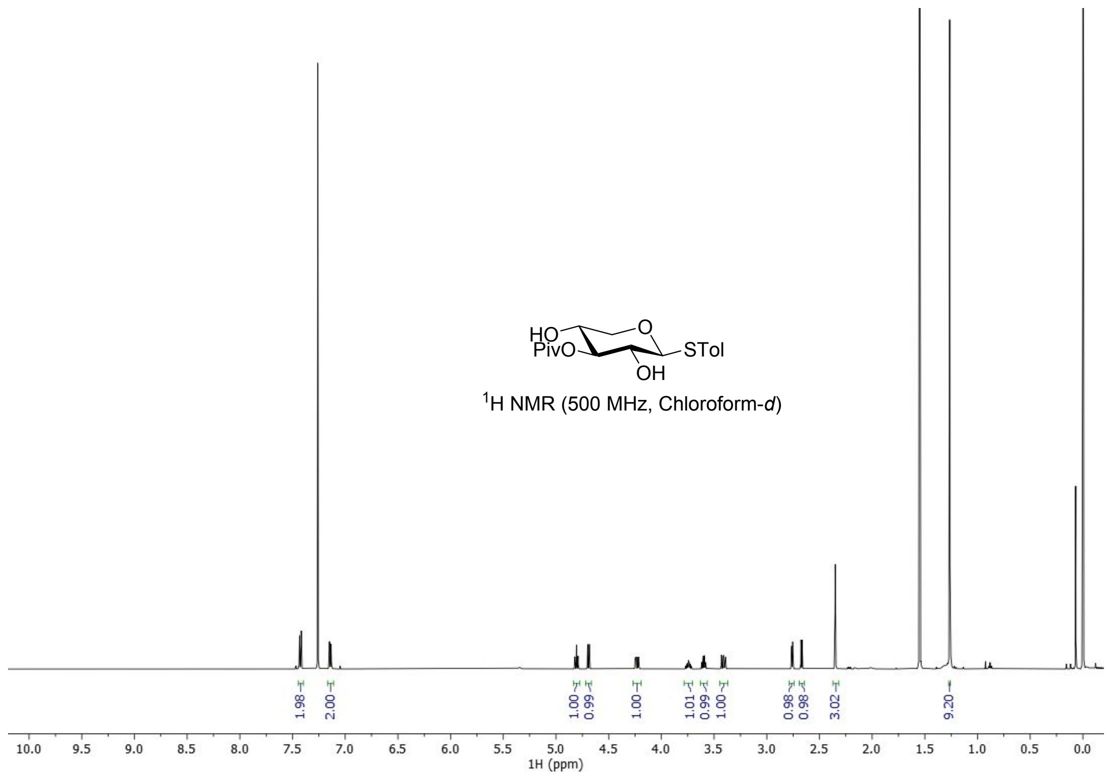
HO
PivO
STol
OH
^{1}H NMR (500 MHz, Chloroform-*d*)
1.98
2.00
1.00
0.99
1.00
1.01
0.99
1.00
0.98
0.98
3.02
9.20
1H (ppm)

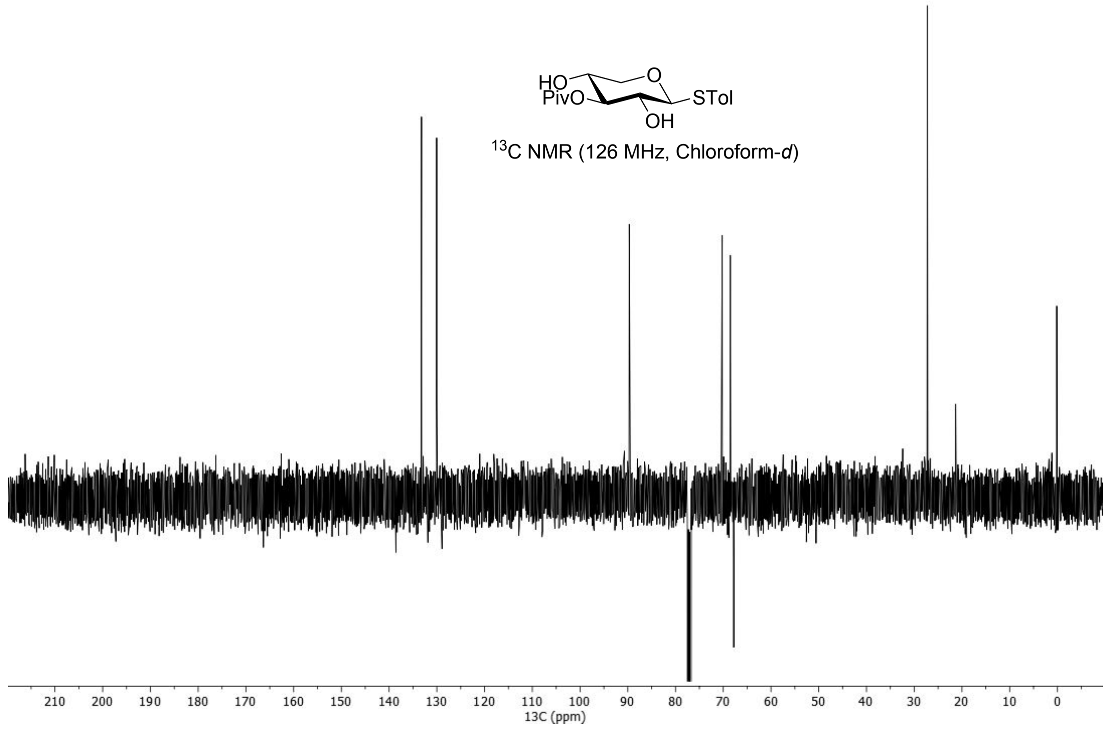
HO
PivO
STol
OH
^{13}C NMR (126 MHz, Chloroform-*d*)
13C (ppm)

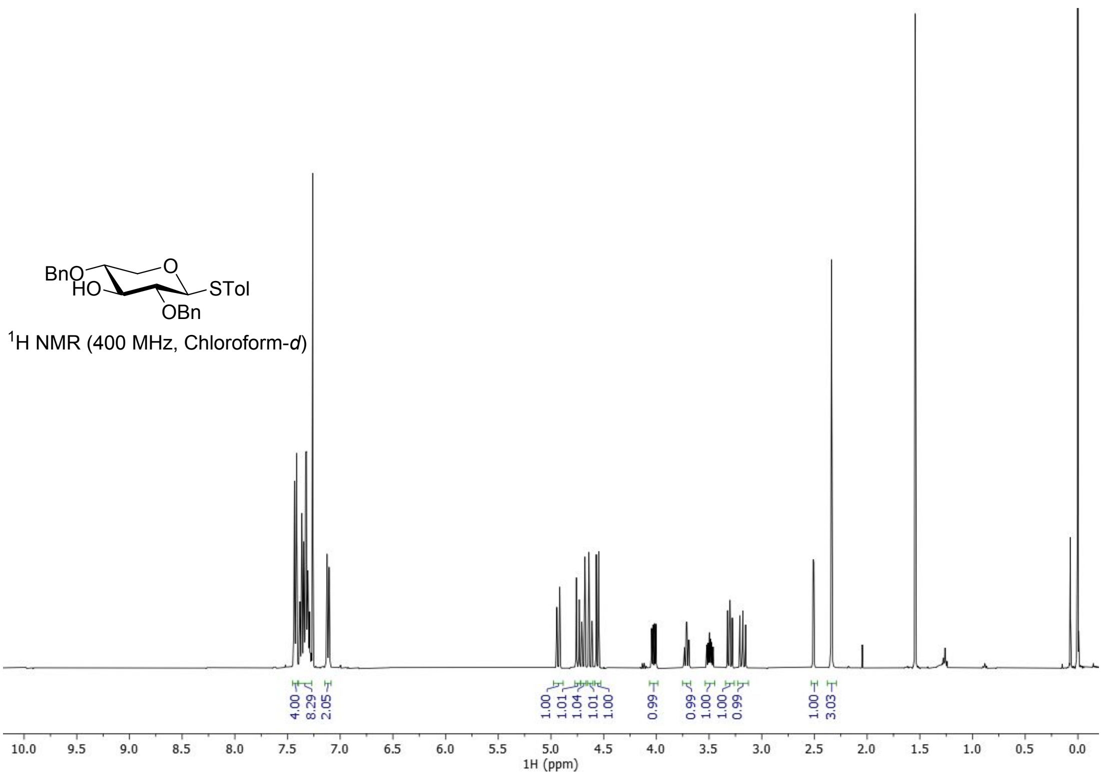
BnO
HO
O
STol
OBn
1H NMR (400 MHz, Chloroform-d)
4.00
8.29
2.05
1.00
1.01
1.04
1.01
1.00
0.99
0.99
1.00
1.00
0.99
1.00
3.03
1H (ppm)

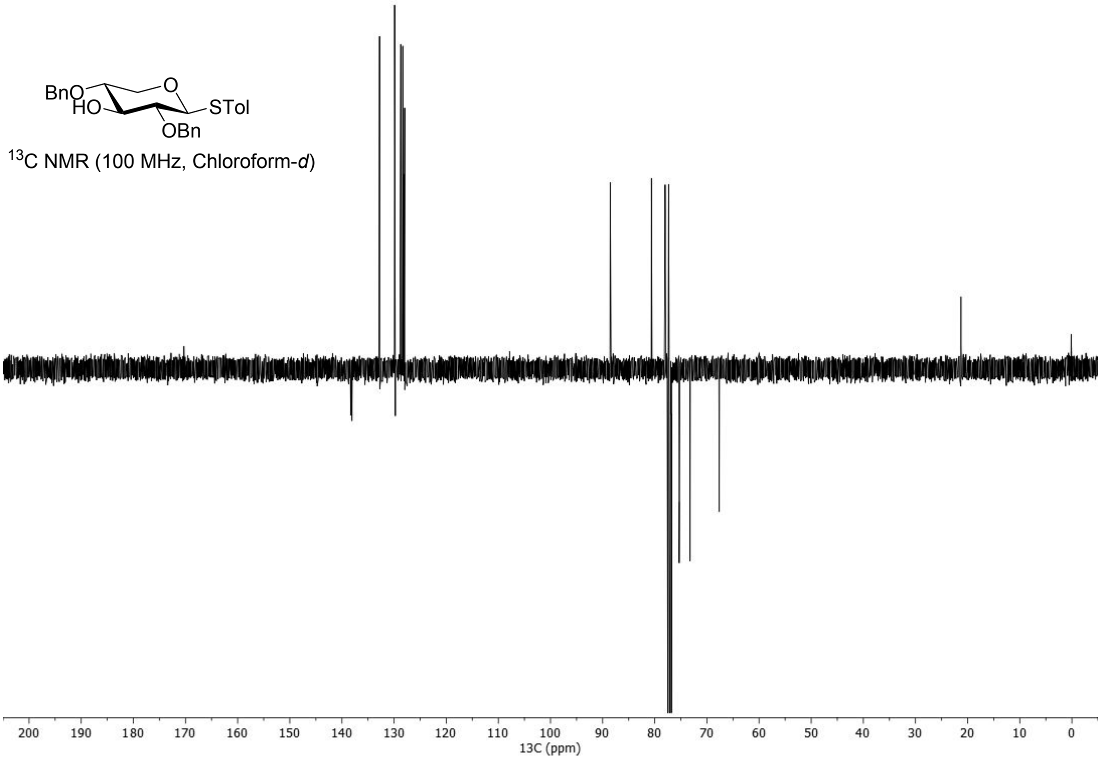
BnO
HO
O
STol
OBn
13C NMR (100 MHz, Chloroform-d)
13C (ppm)

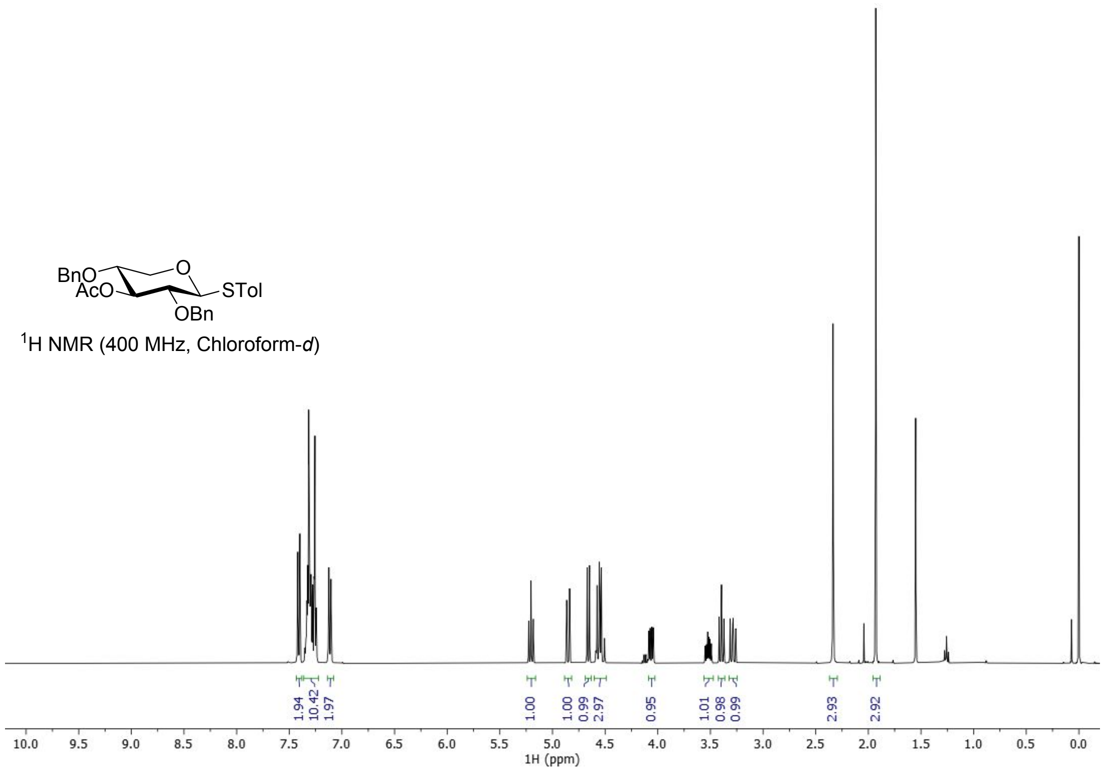
BnO
AcO
O
STol
OBn
1H NMR (400 MHz, Chloroform-d)
1.94
10.42
1.97
1.00
1.00
0.99
2.97
0.95
1.01
0.98
0.99
2.93
2.92
1H (ppm)

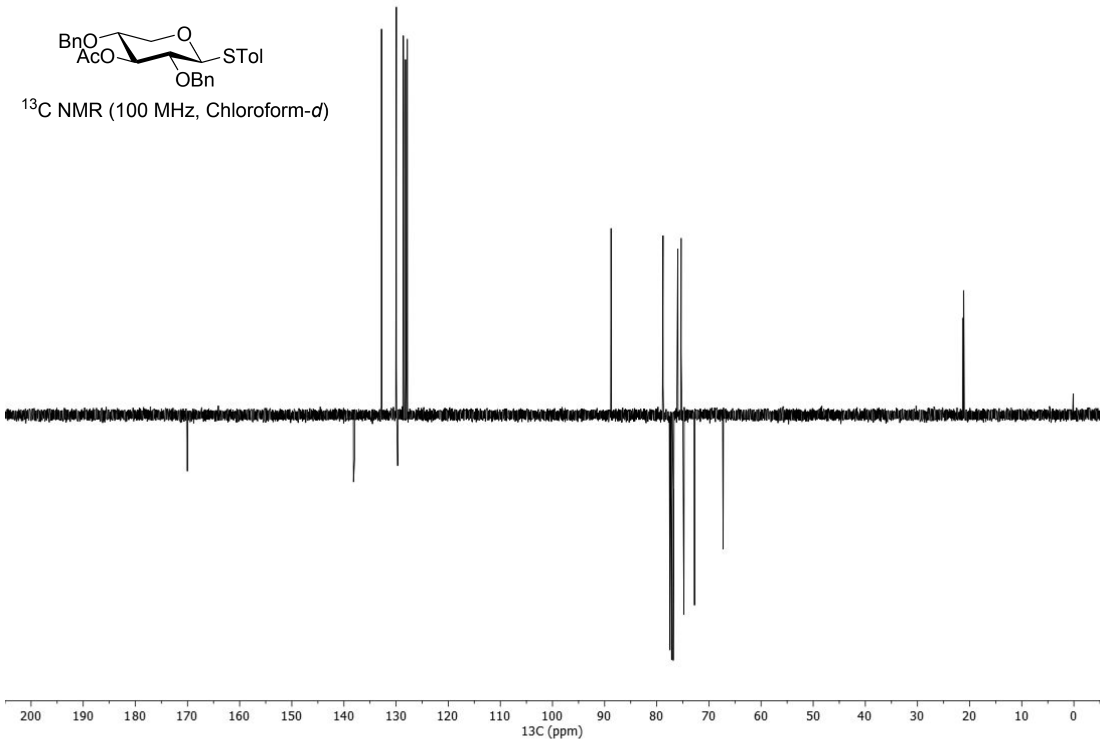
BnO
AcO
O
STol
OBn
13C NMR (100 MHz, Chloroform-d)
13C (ppm)

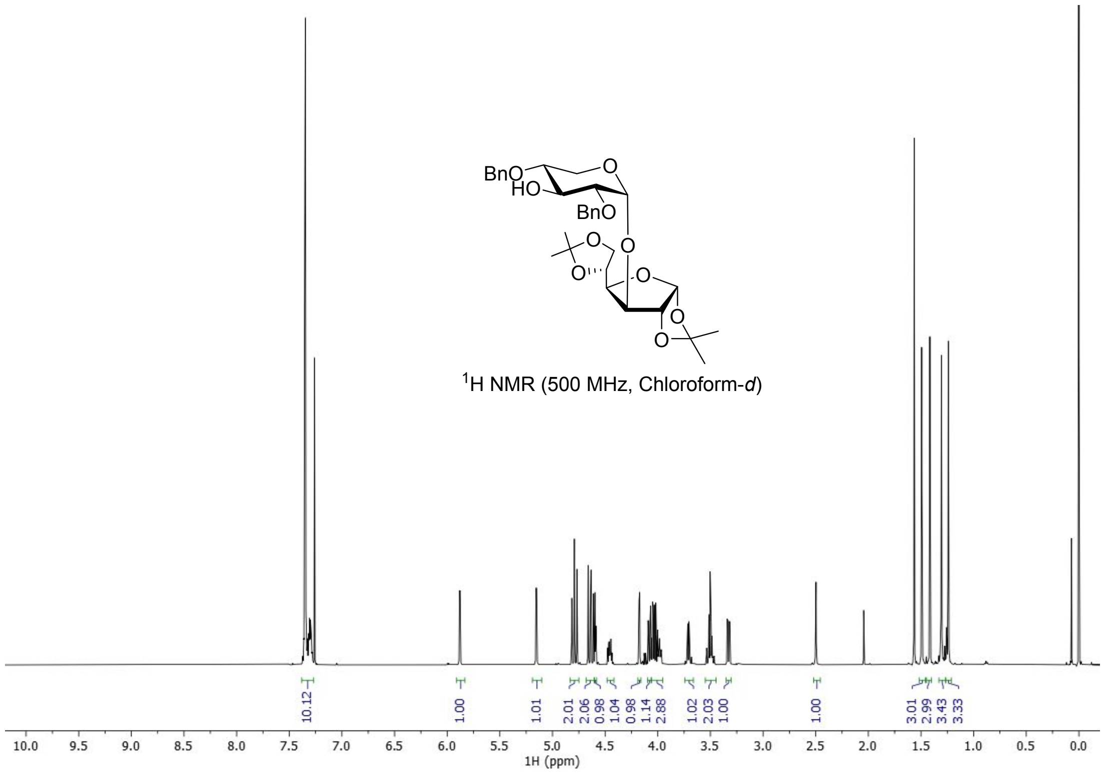
BnO
HO
BnO
O
^{1}H NMR (500 MHz, Chloroform-*d*)
10.12
1.00
1.01
2.01
2.06
0.98
1.04
0.98
1.14
2.88
1.02
2.03
1.00
1.00
3.01
2.99
3.43
3.33
10.0 9.5 9.0 8.5 8.0 7.5 7.0 6.5 6.0 5.5 5.0 4.5 4.0 3.5 3.0 2.5 2.0 1.5 1.0 0.5 0.0
1H (ppm)

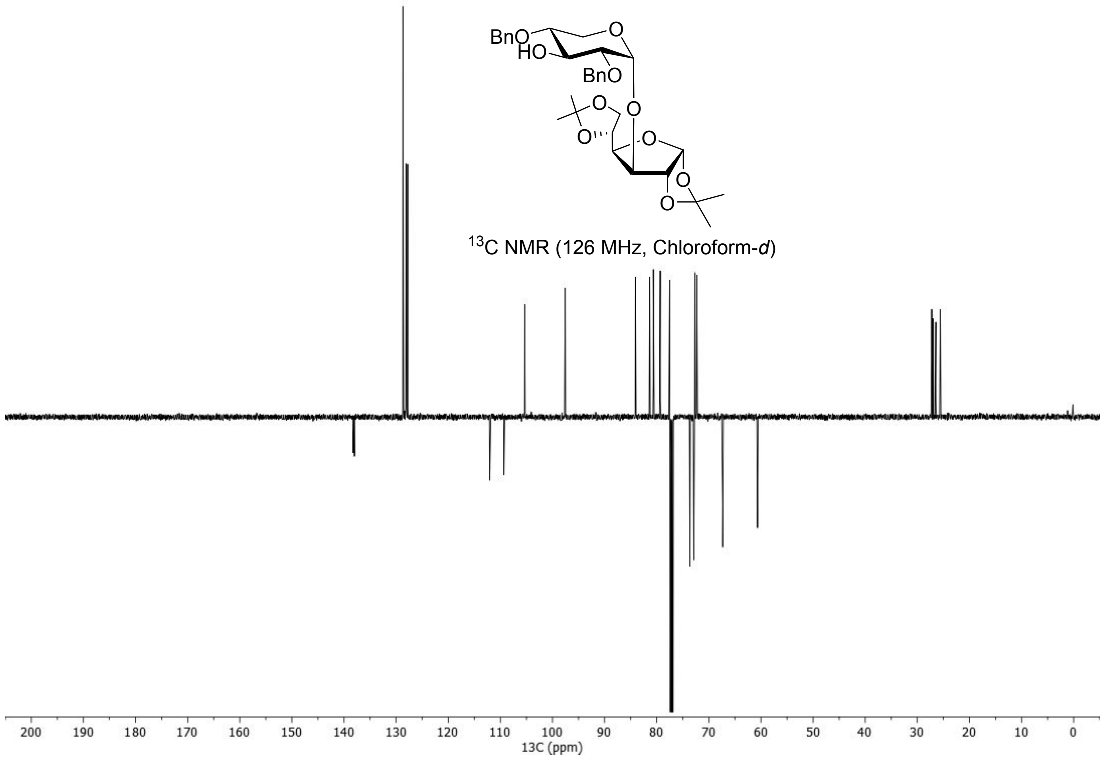
BnO
HO
BnO
O
^{13}C NMR (126 MHz, Chloroform-*d*)
200 190 180 170 160 150 140 130 120 110 100 90 80 70 60 50 40 30 20 10 0
13C (ppm)

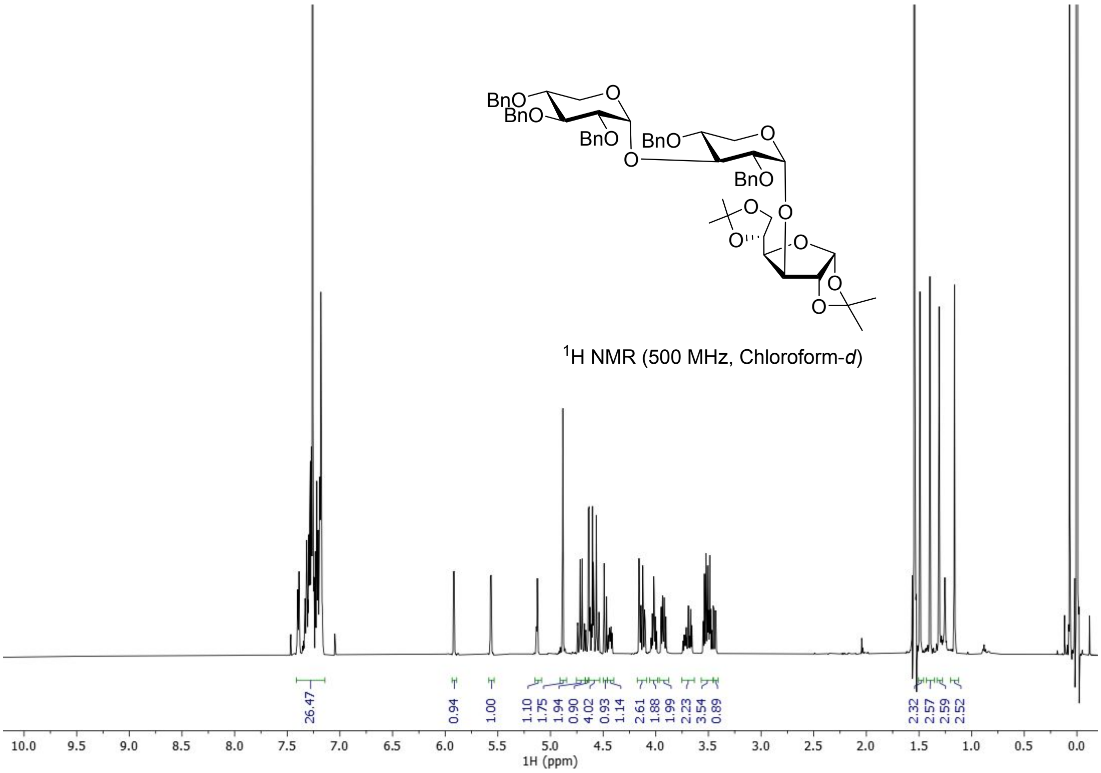
BnO
BnO
BnO
O
BnO
O
BnO
O
O
O
O
O
O
1H NMR (500 MHz, Chloroform-d)
26.47
0.94
1.00
1.10
1.75
1.94
0.90
4.02
0.93
1.14
2.61
1.88
1.99
2.23
3.54
0.89
2.32
2.57
2.59
2.52
10.0 9.5 9.0 8.5 8.0 7.5 7.0 6.5 6.0 5.5 5.0 4.5 4.0 3.5 3.0 2.5 2.0 1.5 1.0 0.5 0.0
1H (ppm)

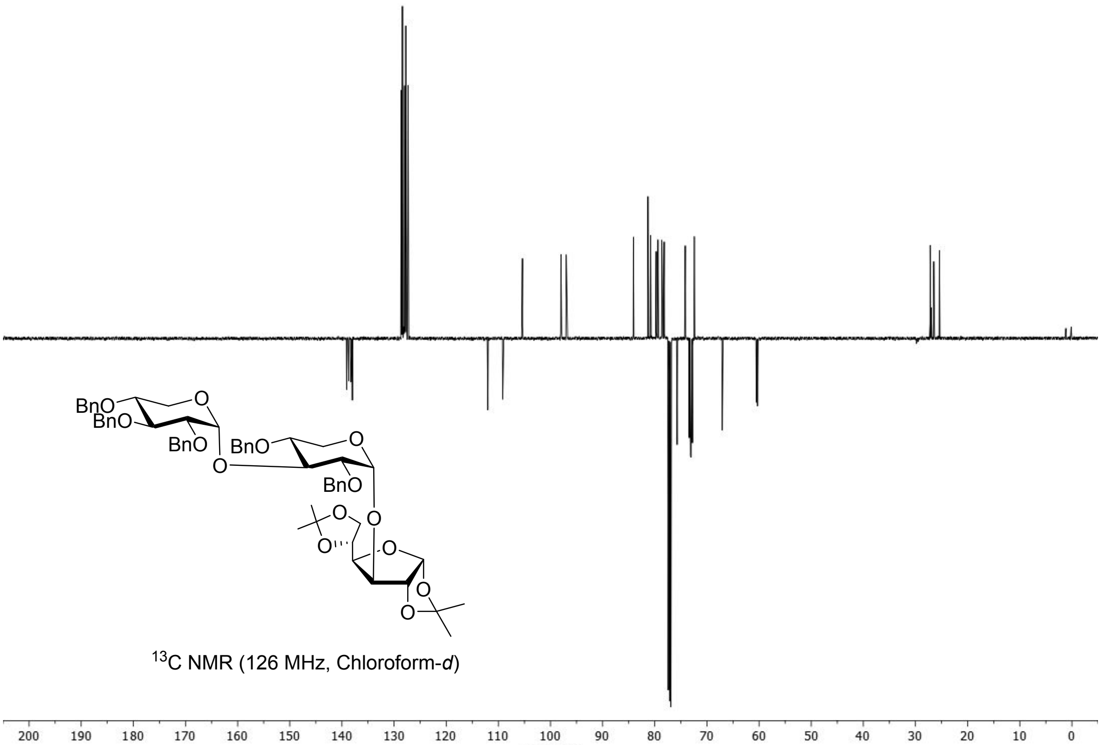
BnO
BnO
BnO
O
BnO
O
BnO
O
O
O
O
O
O
13C NMR (126 MHz, Chloroform-d)
200 190 180 170 160 150 140 130 120 110 100 90 80 70 60 50 40 30 20 10 0
13C (ppm)

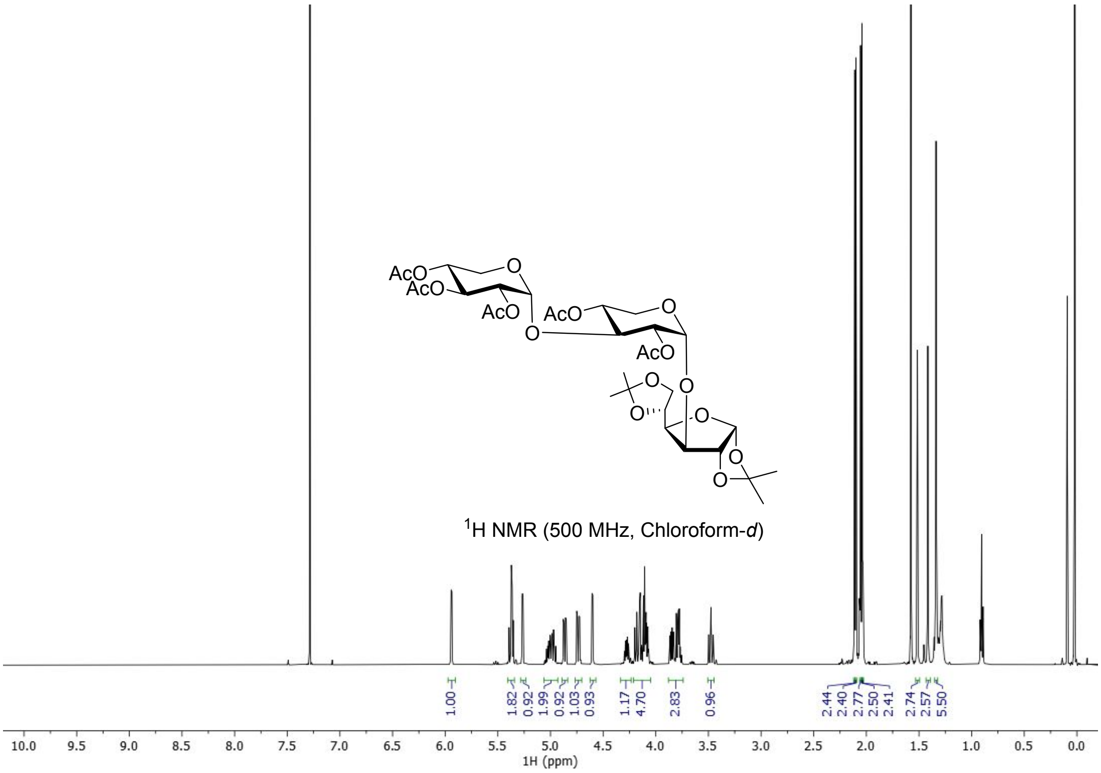

AcO
AcO
AcO
O
O
AcO
AcO
O
O
O
O
O
O
O
1H NMR (500 MHz, Chloroform-d)
1.00
1.82
0.92
1.99
0.92
1.03
0.93
1.17
4.70
2.83
0.96
2.44
2.40
2.77
2.50
2.41
2.74
2.57
5.50
10.0 9.5 9.0 8.5 8.0 7.5 7.0 6.5 6.0 5.5 5.0 4.5 4.0 3.5 3.0 2.5 2.0 1.5 1.0 0.5 0.0
1H (ppm)

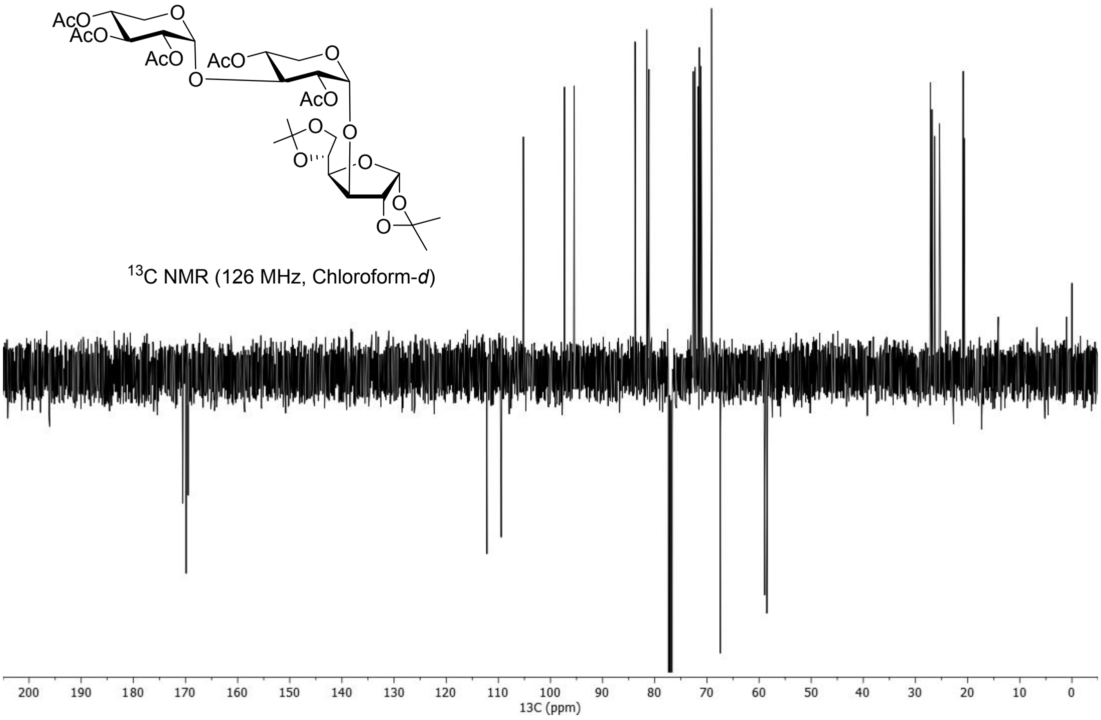

AcO
AcO
AcO
O
O
AcO
AcO
O
O
O
O
O
O
O
13C NMR (126 MHz, Chloroform-d)
200 190 180 170 160 150 140 130 120 110 100 90 80 70 60 50 40 30 20 10 0
13C (ppm)

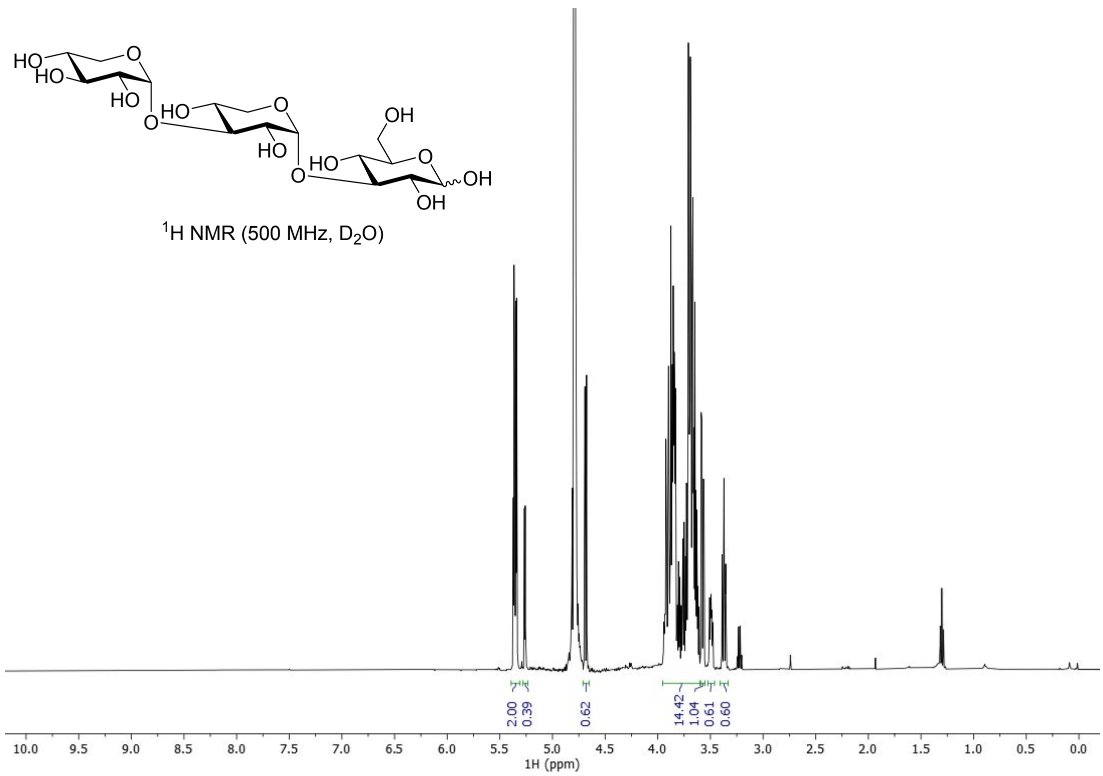

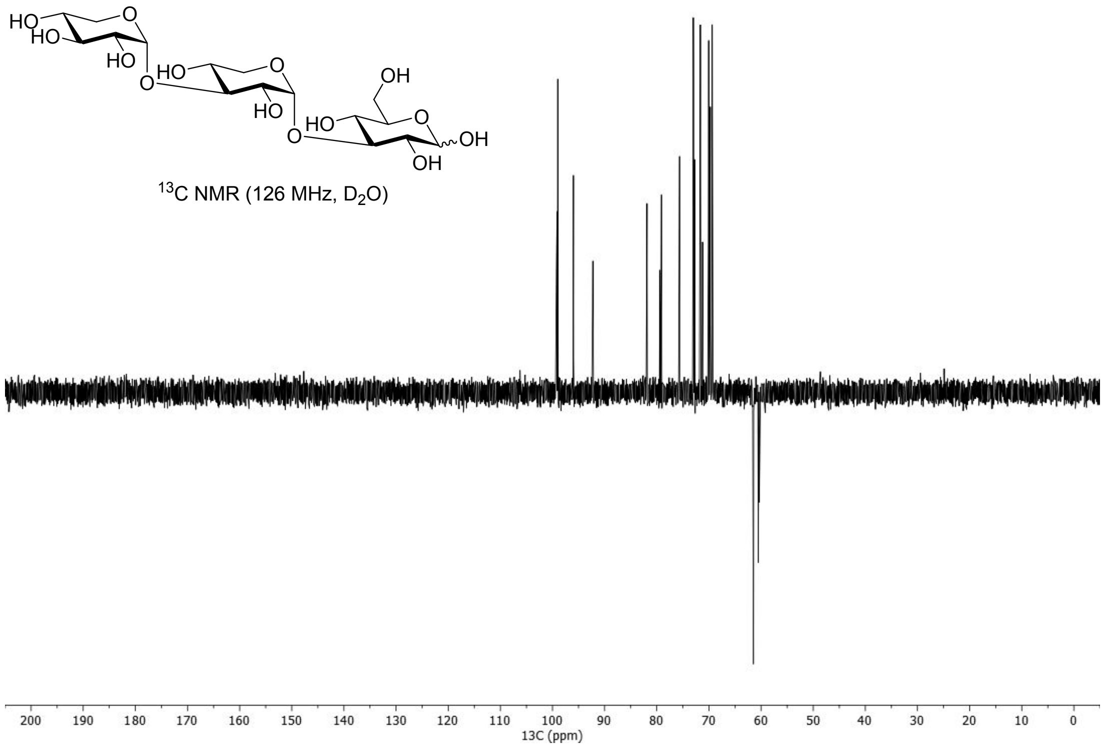

1. De Praeter, C. M.; Gerwig, G. J.; Bause, E.; Nuytinck, L. K.; Vliegenthart, J. F. G.; Breuer, W.; Kamerling, J. P.; Espeel, M. F.; Martin, J.-J. R.; De Paepe, A. M.; Chan, N. W. C.; Dacremont, G. A.; Van Coster, R. N., A Novel Disorder Caused by Defective Biosynthesis of

N-Linked Oligosaccharides Due to Glucosidase I Deficiency. *The American Journal of Human Genetics* **2000,** *66* (6), 1744-1756.

2. Jansen, G.; Muskiet, F. A. J.; Schierbeek, H.; Berger, R.; van der Slik, W., Capillary gas chromatographic profiling of urinary, plasma and erythrocyte sugars and polyols as their trimethylsilyl derivatives, preceded by a simple and rapid prepurification method. *Clinica Chimica Acta* **1986,** *157* (3), 277-293.

3. Berden, G.; Derksen, M.; Houthuijs, K. J.; Martens, J.; Oomens, J., An automatic variable laser attenuator for IRMPD spectroscopy and analysis of power-dependence in fragmentation spectra. *International Journal of Mass Spectrometry* **2019,** *443*, 1-8.

4. Frisch, M. J.; Trucks, G. W.; Schlegel, H. B.; Scuseria, G. E.; Robb, M. A.; Cheeseman, J. R.; Scalmani, G.; Barone, V.; Petersson, G. A.; Nakatsuji, H.; Li, X.; Caricato, M.; Marenich, A. V.; Bloino, J.; Janesko, B. G.; Gomperts, R.; Mennucci, B.; Hratchian, H. P.; Ortiz, J. V.; Izmaylov, A. F.; Sonnenberg, J. L.; Williams-Young, D.; Ding, F.; Lipparini, F.; Egidi, F.; Goings, J.; Peng, B.; Petrone, A.; Henderson, T.; Ranasinghe, D.; Zakrzewski, V. G.; Gao, J.; Rega, N.; Zheng, G.; Liang, W.; Hada, M.; Ehara, M.; Toyota, K.; R. Fukuda, R.; Hasegawa, J.; Ishida, M.; Nakajima, T.; Honda, Y.; Kitao, O.; Nakai, H.; Vreven, T.; Throssell, K.; Montgomery Jr., J. A.; Peralta, J. E.; Ogliaro, F.; Bearpark, M. J.; Heyd, J. J.; Brothers, E. N.; Kudin, K. N.; Staroverov, V. N.; Keith, T. A.; Kobayashi, R.; Normand, J.; Raghavachari, K.; Rendell, A. P.; Burant, J. C.; Iyengar, S. S.; Tomasi, J.; Cossi, M.; Millam, J. M.; Klene, M.; Adamo, C.; Cammi, R.; Ochterski, J. W.; Martin, R. L.; Morokuma, K.; Farkas, O.; Foresman, J. B.; Fox, D. J., Gaussian 16, Revision A. 03. Gaussian, Inc. Wallingford, CT: 2016.

5. van Outersterp, R. E.; Houthuijs, K. J.; Berden, G.; Engelke, U. F.; Kluijtmans, L. A. J.; Wevers, R. A.; Coene, K. L. M.; Oomens, J.; Martens, J., Reference-standard free metabolite identification using infrared ion spectroscopy. *International Journal of Mass Spectrometry* **2019,** *443*, 77-85.
